# The complex build algorithm to set up starting structures of lanthanoid complexes with stereochemical control for molecular modeling

**DOI:** 10.1038/s41598-021-99525-0

**Published:** 2021-11-02

**Authors:** Gabriel H. L. Munguba, Gabriel A. Urquiza-Carvalho, Frederico T. Silva, Alfredo M. Simas

**Affiliations:** Departamento de Química Fundamental, CCEN, Universidade Federal de Pernambuco, Recife, Pernambuco 50670-901 Brazil

**Keywords:** Computational chemistry, Coordination chemistry, Inorganic chemistry, Materials chemistry

## Abstract

When handling metallic centers of higher coordination numbers, one is commonly deluded with the presumption that any assembled metal complex geometry (including a crystallographic one) is good enough as a starting structure for computational chemistry calculations; all oblivious to the fact that such a structure is nothing short of just one out of several, sometimes dozens, or even thousands of other stereoisomers. Moreover, coordination chirality, so frequently present in complexes of higher coordination numbers, is another often overlooked property, rarely recognized as such. The Complex Build algorithm advanced in this article has been designed with the purpose of generating starting structures for molecular modeling calculations with full stereochemical control, including stereoisomer complete identification and coordination chirality recognition. Besides being in the chosen correct stereochemistry, the ligands are positioned by the Complex Build algorithm in a very unobstructed and unclogged manner, so that their degrees of freedom do not hinder or even choke one another, something that would otherwise tend to lead to negative force constants after further geometry optimizations by more advanced computational model chemistries. The Complex Build algorithm has been conceived for any metallic center, but at present is targeting primarily lanthanoids whose coordination numbers range mostly from 5 to 12 and often lead to a combinatorial explosion of stereoisomers.

## Introduction

Lanthanoid complexes display a myriad of applications: in luminescence sensors and probes^1,2^, in solid-state components with tunable light emissions^2,3^, in electroluminescent materials^4^, in thermosensing devices^5^, as contrast agents in magnetic resonance imaging^6^, in molecular magnets^7^, nanomagnets^8^, as well as in many others^9^.

Metal complexes are capable of displaying a multitude of coordination numbers, CNs, ranging from the extremes of one^10^ up to the very high number of sixteen^11^. Transition metal complexes display CNs mostly in the range of 2–9, with six being the most common. Coordination isomerism in transition metal complexes is definitely important, as exemplified by the two stereoisomers of complex diamminedichloroplatinum(II), commonly referred to as cisplatin and transplatin. Cisplatin is an intravenous chemotherapy medication, used in the treatment of a variety of cancers, whereas its isomer, transplatin, has none of that activity^12,13^.

Lanthanoid complexes display greater CNs, mostly ranging from 6 to 12 as a result of their larger ionic radii^14^. Moreover, the lack of directionality of the lanthanoid coordination bonds, because of their essentially electrostatic nature, leaves almost entirely with the ligands the responsibility of randomly approaching and sterically accommodating themselves around the ionic metal center^15^. This gives rise to a multitude of possible shapes for their coordination polyhedra that lead to a combinatorial explosion of different conceivable stereoisomers, including multiple enantiomeric pairs, with each one, in principle, being perfectly adequate to be used as a starting geometry to be subsequently relaxed by energy-based molecular modeling computational chemistry calculations.

However, how to choose a realistic initial configuration, or how to generate a complete set of stereochemically controlled starting geometries of a lanthanoid complex—a task which is key to success in molecular modeling—is actually far from trivial.

As is the case of the asymmetric carbon, a tetrahedral metal complex connected to four different ligands is a stereogenic center, giving rise to two distinct stereoisomers—a pair of enantiomers—each one a non-superimposable mirror image of the other. Of course, metal ions may also be stereogenic centers in complexes of greater coordination numbers, with the potential of displaying a vastly richer stereochemistry. As exemplified in Table 1, the number of stereoisomers of metal complexes with all different ligands, and thus necessarily chiral, for the prevalent shapes, explodes combinatorially from only 2, for the T-4 tetrahedron shape of CN 4; to 30 for the OC-6 octahedron shape of CN 6; to 5040, for the very common (in lanthanoid complexes) SAPR-8 square antiprism of CN 8; and up to a staggering 453,600 stereoisomers for the JBCSAPR-10 bicapped square antiprism of CN 10^16^.

**Table 1:** Numbers of stereoisomers (all chiral) for metallic complexes with all different monodentate ligands, of coordination numbers, CNs, from 4 to 10, for prevalent polyhedral shapes.

Coordination number	Polyhedral shape symbol	Polyhedral shape name	Number of stereoisomers^16^
4	T-4	Tetrahedron	2
5	TBPY-5	Trigonal bipyramid	20
6	OC-6	Octahedron	30
7	PBPY-7	Pentagonal bipyramid	504
8	SAPR-8	Square antiprism	5040
9	TCTPR-9	Tricapped trigonal prism	60,480
10	JBCSAPR-10	Bicapped square antiprism	453,600

For CNs greater than 4, even when there are repeating ligands, there can be chirality and a multitude of stereoisomers^16^. For example, a lanthanoid complex of CN-8, such as triaquabis(vanillin-O,O)nitritoerbium(III)^17^, ${\text {Er}}({\text {C}}_8{\text {H}}_7{\text {O}}_3)_2({\text {NO}}_2)({\text {H}}_2{\text {O}})_3$, of shape BTPR-8 (biaugmented trigonal prism), could have, in principle, 640 different possible stereoisomers, of which 628 would be chiral (314 pairs of enantiomers) and 12 would be achiral. An even simpler and representative complex of CN-9, triaquatrinitro-Lu(III)^18^, ${\text {Lu}}({\text {NO}}_3)_3({\text {H}}_2{\text {O}})_3$, of shape MFF-9 (muffin), has 232 different possible stereoisomers, of which 222 are chiral (111 pairs of enantiomers) and 10 are achiral^16^.

The high number of stereoisomers of lanthanoid complexes, prompted us to recently classify them by shape and, within each shape, into subsets of same coordination point group symmetry; also advancing the concept of random coordination ratios, the “relative probabilities of occurrence of subsets of stereoisomers of same-symmetry point groups in the limiting situation when energetic effects are equivalent”^16^.

Apart from a crystallographic determined geometry, the actual detailed three-dimensional structure of a lanthanoid complex in the solid state is usually unknown. And if the lanthanoid complex is in solution, there is no guarantee that its shape and stereoisomer will be the same as in a crystal^19^. Actually, what is usually known about a complex is its molecular formula, the formulas of the ligands, and, perhaps, due to some additional experimental evidence, the point group symmetry of the coordination polyhedron and/or its presumed shape. Even in such cases, many structural possibilities still may need to be explored.

Let us consider the case of the above-mentioned complex ${\text {Lu}}({\text {NO}}_3)_3({\text {H}}_2{\text {O}})_3$^18^, a typical one. Let us assume we know from other experiments that, for a given chemical environment, the coordination polyhedron displays a C_s_ symmetry and belongs to the MFF-9 (muffin) shape. Outside of crystallographic results, this is already quite a significant amount of information. Even so, there are still ten achiral stereoisomers possible. Which one of them should be used in further molecular modeling calculations? What if someone wants to take into account all ten possibilities? How can these ten structures be built with a guarantee that they form, without redundancy, a complete set of stereoisomers for this subset? Given the variety of ligand selections and geometric shapes that can generate stereoisomers, it is currently very difficult (if not at all impossible when their numbers are large) to devise by hand all of the spatial dispositions of the ligands around the metallic center, for each possible stereoisomer. Each such stereoisomer would have to be built, one by one, and this work would require mathematical rigor relating to graph and group theory to guarantee that every possible stereoisomer is accounted for.

Common default geometry optimization techniques available in molecular modeling softwares are very sensitive to the starting geometry. Given that the various possible stereoisomers of lanthanoid complexes all represent potential local minima in the configurational energy hypersurface, the resulting geometry often remains trapped within the neighborhood of a given stereoisomer arbitrarily used as a starting configuration. Consequently, since every conceivable stereoisomer may end up in a local minimum, a researcher may need to explore all possibilities. And if there is a correct final geometry to be found, the optimization will only be successful if the starting configuration was sufficiently close to it. In any way, a researcher needs to be aware of all of the stereoisomer possibilities, even if a particular one ends up being chosen. And even in this last case, there is a need for full stereochemical control of the stereoisomer being input, under the penalty of not having a deeper grasp of the structural intricacy of the problem under consideration.

Truly, the quest of constructing starting geometries of metal complexes of high coordination numbers carries a complexity of its own, which goes far beyond that of the tetrahedral carbon atom: a complexity which is totally different from that of building starting geometries of bio-organic molecules, and thus requires a specific and tailored approach of its own.

In this article, we introduce the Complex Build algorithm, an algorithm designed for the specific construction of, not only individual structures, but also complete sets of starting geometries of lanthanoid complexes with full stereochemical control.

## Methodology

Assembling a reasonable geometry for a Lanthanoid complex is a sophisticated task that depends upon a correct mathematical description of many concepts followed by their translation into computer codes, all ultimately leading to an algorithm. Starting with the chemical formula of the complex of interest, one would first need the structures of the ligands, followed by ligand flexibility recognition and the means to organize possible geometries of the higher coordination complex in full stereochemical control of the resulting coordination polyhedron.

Refining the structure of a complex of higher coordination requires optimization of its geometry with respect to an objective function that encodes empirical measures of quality—measures that must be consistent with what would be expected of a resulting efficient starting structure for further higher-level molecular modeling calculations.

Of course, if such optimization was to take place in the hyperspace of all Cartesian positions of all of its atoms, it would unnecessarily take far too long. Without a doubt, if all atoms were to be left unrestricted and loose, most of the Cartesian hyperspace would contain atom arrangements that would correspond to spurious, not chemically feasible geometries—something that would only add noise to the procedure. Because the problem is intrinsically non-linear and of a very high dimensionality, it would be at least impractical, if not unfeasible, with today’s typical computational capabilities to explore all of the potential hyperspaces of a complete set of several stereoisomers, in numbers that can easily run into the hundreds. That is why we performed many computational experiments to arrive at a simple, yet sufficiently accurate, heuristic objective function. As a result, we devised a different set of coordinates to codify the chemically relevant features of the structures of a metal complex. These coordinates relate to particular degrees of freedom accessible only to the ligands, such as positions of the coordinating atoms in the coordination sphere, rigid rotations of each ligand around its barycenter, wheel angles, hinge angles, and internal torsions within the ligands. In Complex Build, a mathematical description of these degrees of freedom is based on the chemical structural formulae of the complexes. Thus, a good abstract model of the coordinating ligands in the complexes is an essential part of the algorithm. Changes in coordinates correspond to transformations along the above-mentioned degrees of freedom with respect to a specific first structure, which is taken as the origin of this reduced coordinate space. This first structure must be obtained through a process of geometry initialization which produces it so that its vicinity, in the objective function hypersurface defined in the reduced coordinate set, is sufficiently smooth to avoid trapping the optimized geometry into non-physical local minima. This is achieved by using a set of target values for the coordination bonds between lanthanoid ions and the most frequent coordinated atoms, tabulated from a representative lanthanoid metallic complex dataset. These same target values are employed in the knowledge-based objective-function of the optimization process to ensure that the initialized values are sustained throughout the assembly procedure of the starting structure. Finally, the algorithm allows the precise stereochemical control of the output structure.

**Figure 1:**
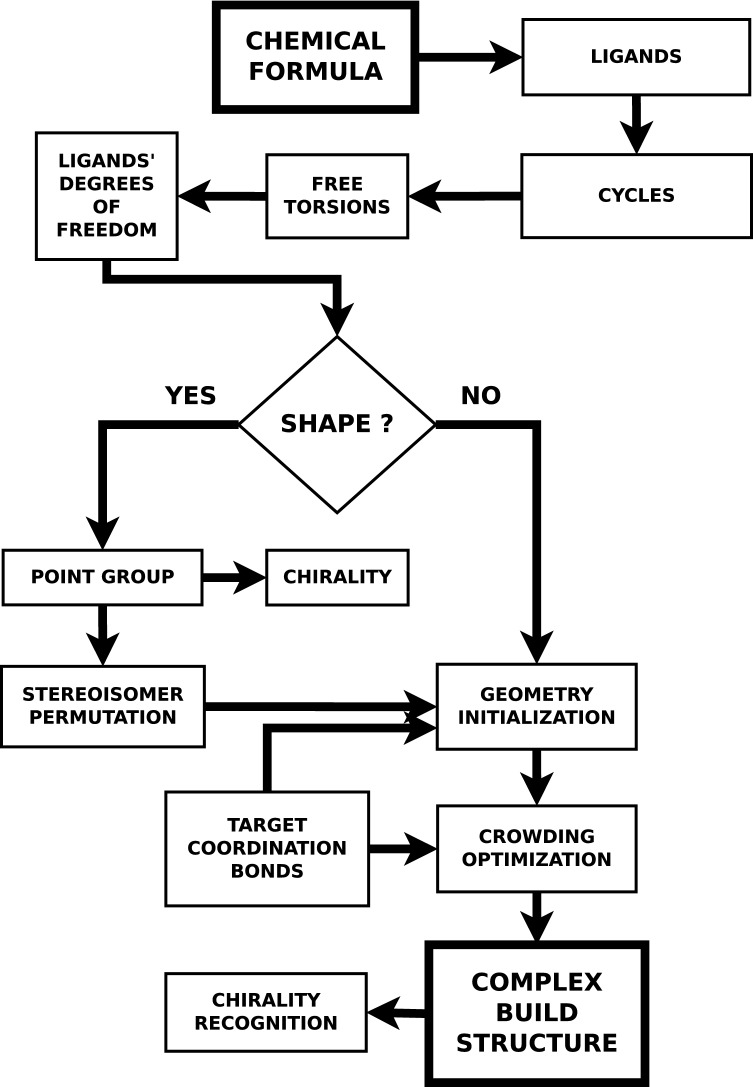
A flowchart describing the hierarchy and links among the various subtasks related to the overall goal of producing, from the chemical formula, 3D structures of a complex that can function as effective starting geometries to be further input into more sophisticated molecular modeling softwares.

Figure 1 presents a flowchart summarizing the various mutually interacting tasks and subtasks needed to first assemble a complex from its chemical formula, and, then, to produce 3D structures, with full stereochemical control, to be subsequently input as effective starting geometries into more sophisticated molecular modeling softwares.

### Notation

Because of this rich stereoisomerism, we need to establish a notation for a generic chemical formula for metallic complexes to be able to express the various coordination polyhedra that one can expect to find in coordination compounds, in order to allow their correct classification and enumeration. As is well known in chemistry, ligands are either molecules, molecular ions, or atomic ions that bind to a central metal atom to form a species called a metal complex. Denticity of a ligand is the number of coordinating atoms of donor groups in a ligand that bind to the central metal atom. Ligands with a single tooth are called monodentate; ligands with two teeth are called bidentate; with three teeth, tridentate, and so on. From the point of view of stereoisomerism control, the important characteristics of a ligand are: (1) its denticity, and (2) when bidentate or of higher denticities, how chemically symmetric or asymmetric it is upon the exchange of any of its coordinating atoms by means of simple molecular rotations.

For the purpose of this article, we will use the same generic formula notation of our previous work^16^, where the metal is represented by “M”, monodentate ligands are represented by lower-case latin letters and polydentate ligands are represented by strings of upper-case latin letters. The letters should represent the chemical variety of each tooth. A symmetric bidentate ligand (one that does not change chemically if the coordinating atoms are switched), for example, would be “AA” in the formula. If we want to express a center bonded to two distinct bidentate symmetric ligands, then it would be M(AA)(BB). On the other hand, an asymmetric bidentate ligand would be represented as (AB). A tetradentate ligand with four identical teeth (such as a porphyrin, for instance) would be (AAAA). Different chemical donor groups must be represented by different case sensitive letters; and one can use as many letters as needed. The number following the ligand symbol indicates the number of times a ligand appears in the chemical formula. The ligands are ordered in terms of their denticities, with monodentates first, followed by bidentates, and so on. Within each denticity they are ordered from the most repeating to the least repeating one. As examples: the complexes mentioned in the introduction, one of chemical formula ${\text {Er}}({\text {C}}_8{\text {H}}_7{\text {O}}_3)_2({\text {NO}}_2)({\text {H}}_2{\text {O}})_3$, is represented by the generic notation by Ma3b(AB)2, and the other, ${\text {Lu}}({\text {NO}}_3)_3({\text {H}}_2{\text {O}})_3$, by Ma3(AA)3.

### Atomic and molecular systems

We define an atomic or molecular system as a collection of atoms joined together by chemical bonds. In the Complex Build notation, such a system ${\mathscr{M}}$ is represented by a set of three elements: ${\mathscr{M}}=\{{\mathscr{A}},\tau ,{\mathbf {X}}\}$; a set of atoms ${\mathscr {A}}$, a topology function $\tau$, which encodes the connectivities of the atoms in ${\mathscr {A}}$, and a geometry matrix ${\mathbf {X}}$.

The $|{\mathscr {A}}| \times 3$ geometry matrix of a system is such that $\text {X}_{n1} = x_n$, $\text {X}_{n2} = y_n$ and $\text {X}_{n3} = z_n$ where $x_n$, $y_n$ and $z_n$ are the coordinates in the Cartesian representation of the *n*-th atom position vector, denoted by ${\mathbf {X}}[n]=\mathbf {p}_n=(x_n,y_n,z_n)$. Each atom in ${\mathscr{A}}$ is associated with the row in ${\mathbf {X}}$ that holds its position vector. This row number serves as a unique serial index for each atom, giving ${\mathscr{A}}$ a list structure in which its elements can be presented as a unique ordered sequence. Alternatively, we might want to refer to the position vector without using its row index, in which case we use ${\mathbf {X}}[a]$ to denote the position vector of a generic atom *a*. This notation is also expanded to sets of atoms such that ${\mathbf{X}}[{\mathscr{a\!}}]$ refers to all of the position vectors of the atoms in the subset ${\mathscr{a\!}}\subset {\mathscr{A}}$ taken as a matrix.

In the Complex Build context, bonds can have one of five allowed bond orders: 0 for no bond, 1 for either single bonds or for coordination bonds, 2 for double bonds, 3 for triple bonds and 1.5 for delocalized double bonds. The topology function $\tau$ maps pairs of atoms into the set of allowed bond orders such that $\tau : {\mathscr{A}}\times {\mathscr{A}}\rightarrow \{0,1,2,3,1.5\}$. The notation $\tau (a,b)$ denotes the bond order between atoms *a* and *b*. By construction, $\tau (a,a)=0$ because atoms are not allowed to form bonds with themselves.

Using the above topology function, we can define the neighborhood of atom *a*, denoted by ${\mathscr{N}}[a]$, as:

1 $$\begin{aligned} {\mathscr{N}}[a] = \{ b \in {\mathscr{a}}| \tau (a,b) \ne 0 \} \end{aligned}$$

Presence in a neighbourhood is a two-way property so if $a\in {\mathscr {N}}[b]$ then $b\in {\mathscr {N}}[a]$. This notation can be extended to sets of atoms. For instance, suppose there exists a set ${\mathscr {a\!}}$ of atoms, then ${\mathscr {N}}[{\mathscr {a\!}}] = \bigcup _{a \in {\mathscr {a\!}}} {\mathscr {N}}[a]$. One consequence of the above definitions is that:

2 $$\begin{aligned} \tau (a,b) = 0 \forall b \notin {\mathscr {N}}[a] \end{aligned}$$

An angle $\theta$ is a set of three atoms such that one of them, the vertex of the angle, is connected to the other two, the sides of the angle. However, the sides are not necessarily connected to one another. This statement can be summarized in Eq. (3)

3 $$\begin{aligned} \theta = \{s_1,v,s_2 \in {\mathscr {A}}| s_1,s_2 \in {\mathscr {N}}[v] \wedge s_1 \notin {\mathscr {N}}[s_2]\} \end{aligned}$$

in which, atoms $s_1$ and $s_2$ are the sides of the angle and *v*, its vertex.

Let us now consider four atoms $e_1,i_1,i_2$ and $e_2$, such that $e_1$ is bonded to $i_1$, which is in turn bonded to $i_2$, which is finally bonded to $e_2$. $e_1, i_1$ and $i_2$ form a plane. Likewise, $i_1$, $i_2$ and $e_2$ form another plane. The dihedral angle $\phi$ is the angle formed between the two planes at their intersection along the bond between the atoms they have in common, $i_1$ and $i_2$, that also defines the dihedral torsion axis. $i_1$ and $i_2$ are thus the internal atoms of the dihedral and $e_1$ and $e_2$ are the external ones. In the sense of Eq. (3), we define a dihedral angle according to Eq. (4)

4 $$\begin{aligned} \phi = \{ e_1,i_1,i_2,e_2 \in {\mathscr {A}}| e_1,i_2 \in {\mathscr {N}}[i_1] \wedge e_1 \notin {\mathscr {N}}[i_2] \wedge e_2 \in {\mathscr {N}}[i_2] \wedge e_2 \notin {\mathscr {N}}[e_1] \cup {\mathscr {N}}[i_1] \} \end{aligned}$$

where $i_1$ and $i_2$ are the internal atoms and $e_1$ and $e_2$ external ones.

Any four atoms connected, so as to obey the definition in Eq. (4), form a valid dihedral. However, not all existing dihedral angles may correspond to degrees of freedom in our Complex Build algorithm. Only those that, when varied simultaneously, do not modify any of the bond angles within each of the ligands; that is, excluding bond angles involving the central metal ion. Since torsions around a dihedral take place around its axis, any dihedrals sharing the same internal atoms define rotations around the same axis. When a torsion is applied around an axis, all of the dihedrals that share this same axis are torsioned by the same angle. Because all of these rotations are necessarily correlated, only one of them can be a degree of freedom in our Complex Build algorithm. As such, for the purposes of defining degrees of freedom available to a complex, two dihedrals will be the same if their internal atoms are the same.

This allows us to state that any two atoms $i_1$ and $i_2$ define a dihedral if there is at least one element $e_1 \in {\mathscr {N}}[i_1]$ and another $e_2 \in {\mathscr {N}}[i_2]$ such that they satisfy Eq. (4) as if $i_1$ and $i_2$ were the internal atoms, and $e_1$ and $e_2$ the external ones. As a convention, we can define a unique degree of freedom dihedral between $i_1$ and $i_2$ by requiring that $e_1$ be the element in ${\mathscr {N}}[i_1]$ with the lowest serial-index that obeys the relationship in Eq. (4), and likewise for $e_2$. The list of dihedral angles defined as above does not account for every single possible dihedral, but only for those that represent conformational degrees of freedom in the context of the Complex Build algorithm. We refer to these as proper dihedrals.

### Ligands and complexes

Ligands are a type of atomic or molecular system ${\mathscr {M}}$. A ligand ${\mathscr {l}}$ that extends a system ${\mathscr {M}}$ is given as ${\mathscr{l}}=\{{\mathscr{t\!}}\} \cup {\mathscr {M}}$. Where ${\mathscr{t\!}}\subset {\mathscr {A}}$ is a special set of atoms called the teeth of the ligand. Complexes are also a type of molecular system ${\mathscr {M}}$. A complex ${\mathscr {C}}$, which is an extension of the molecular system ${\mathscr {M}}$, is denoted by ${\mathscr {C}}= {\mathscr {M}}\cup \{{\mathscr{L}},c,T\}$. The complex also has a special atom $\text {Me}\in {\mathscr {A}}$ called its metallic center, placed at the origin of the coordinate system such that ${\mathbf {X}}[\text {Me}]=\mathbf {0}$. The set ${\mathscr{L}}= \{{\mathscr{l}}_n\}$ is the set of the ligands of the complex. The atom set, topology function, and teeth set of the *n*-th ligand in the complex, are referred to as ${\mathscr {A}}_n$, $\tau _n$, and ${\mathscr{t}}_n$, respectively. Consequently:

5 $$\begin{aligned} {\mathscr {A}}= \{\text {Me}\} \cup \left[ \bigcup _k^{|{\mathscr{L}}|} {\mathscr {A}}_k \right] \end{aligned}$$

In a complex, the topology around the metallic center is special. The metal is only allowed to be connected to the teeth of its ligands and to no other atom, such that:

6 $$\begin{aligned} \Pi = {\mathscr {N}}[\text {Me}]=\bigcup _n^{|{\mathscr{L}}|}{\mathscr{t}}_n \end{aligned}$$

The bonds between the metallic center and its neighbors are the coordinate bonds of the complex. The cardinality of the neighbourhood of the metallic center, $|\Pi |$, is the coordination number of the complex. Coordinate bonds always have a bond order of 1, a definition that can be symbolically stated as:

7 $$\begin{aligned} \tau (\text {Me},b) = 1 \forall b \in {\mathscr {N}}[\text {Me}] \end{aligned}$$

The set $\Pi$ is called the coordinate system of a complex. The particular spatial disposition of the atoms in the coordinate system forms the coordination polyhedron. The regularity and shape of such polyhedra are observables and determine many chemical properties of these species, particularly photochemical properties.

The complex also has a function $c: \Pi \rightarrow {\mathbb {N}}$ which we call the *color* of each coordinated tooth. Each tooth of a ligand has a color, which encodes its chemical environment. Two teeth with the same color, for instance, must have the same chemical environment, therefore they must be bound to the same types of atoms with the same bond orders. The atoms bound to each other must in turn also be bound to an identical group of atoms, via the same bond orders, and so on, and so forth. The consequence of this is that similarity of teeth colors often correlates with topological symmetries in the molecular structure of the complex, such as multiple instances of the same type of ligand, or unusually symmetric ligands.

We also define the function $T_n:{\mathscr{L}}\rightarrow {\mathbb {N}}^{|{\mathscr{t}}_n|}$ which we call the type of the ligand. The type of a ligand is a tuple in which each element is the color of one of the ligand’s teeth, ordered in accordance to the serial index of each tooth in the structure.

The ligands in ${\mathscr{L}}$ are organized in ascending order of their denticities and colors and descending order of the number of equal colors in their types.

These criteria are applied following this specific priority order. First, the list is ordered according to the denticity, then, each batch of ligands with equal denticity is compared according to their colors. If two ligands are monodentate, then the one with the lowest color in its tooth will come first in the list. Otherwise, the two ligands are first sorted by the number of identically-colored teeth, the one with the largest number comes first. Then, they are compared according to the lowest color in their type. Identical ligands will necessarily be placed beside each other in the list.

This order determines the geometry matrix of the complex ${\mathscr {C}}$ such that ${\mathscr{L}}\in {\mathscr {C}}$.This happens because the geometry matrix of the complex is built as a column-wise concatenation of the geometry matrices of each ligand ${\mathscr{l\!}}\in {\mathscr{L}}$, plus the position vector of the metallic center, such that:

8 $$\begin{aligned} {\mathbf {X}}= \begin{bmatrix}  \vec{\mathbf {0}}\\ {\mathbf {X}}_1 \\ {\mathbf {X}}_2 \\ \vdots \\ {\mathbf {X}}_m \\ \vdots \\ {\mathbf {X}}_{|{\mathscr{L}}|} \\ \end{bmatrix} \end{aligned}$$

Because every atom in a ligand is also an atom in the complex, the same atom will be referenced by two different sets of indices. One set refers to the geometry matrix of the complex, and the other to the geometry matrix of the ligand to which the atom belongs.

The relationship between the geometry matrices of the ligands and of the complex, shown in Eq. (8), establishes a relationship between these two sets of indices. The *i*-th atom in the *m*-th ligand refers to the same *j*-th atom in a complex if the two indices satisfy Eq. (9):

9 $$\begin{aligned} j = i+ \sum _{n<m}^{|{\mathscr{L}}|} |{\mathscr {A}}_n| \end{aligned}$$

Whenever a new notation is introduced that defines some property of the complexes in terms of their associated ligands, it is assumed that such indicial transformations have taken place accordingly.

Likewise, transformations defined in terms of the complex geometry matrix are assumed to have updated each of the corresponding ligand geometry matrices accordingly, such that the geometry of the complex is a composition of the individual geometries of each ligand.

### Cycles

In the Complex Build algorithm, it is assumed that the stereoisomerism of cycles remains the same throughout the assembly process. This has the consequence that the dihedrals within a cycle are not to be considered part of the degrees of freedom that determine the search space for the algorithm.

Since we already have a definition for dihedrals, it is imperative that we now turn to find eventual cycles in the molecule. With the set of cycles and a set of proper dihedrals, it is easy to determine which dihedrals are true degrees of freedom for the purpose of the Complex Build algorithm.

Accordingly, we define the sorted neighborhood ${\mathscr {N}}^*[a,o]$ of a as a list of elements organized in ascending order of the distances $r_{ao} = |{\mathbf {X}}[a]-{\mathbf {X}}[o]|$. Cycles here are represented as sequences of atoms in which every atom is bonded to its successor and predecessor. The last atom in the sequence is thus bonded to the first, and vice-versa. The pseudocode for the computation of the list of cycles is presented in Algorithm 1. 
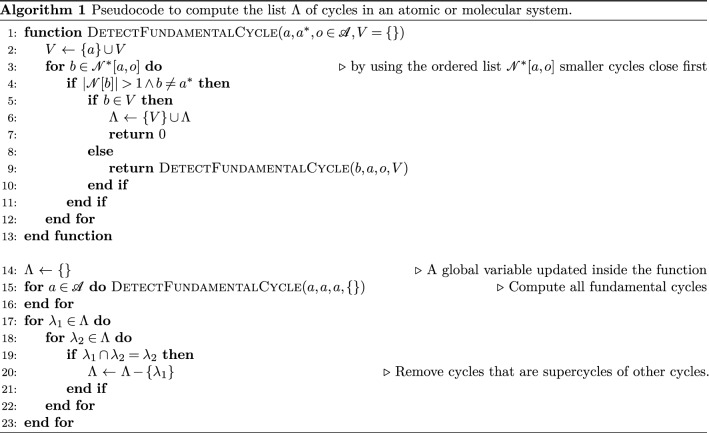


### Ligand degrees of freedom

The final geometry matrix of the complex ${\mathbf {X}}_0$ is the desired output of the Complex Build algorithm. In order to find it, a search must be carried out in the space of subsets of such geometry matrices. The search space accessible geometries are determined by the degrees of freedom available to each ligand.

Fundamentally, in the Complex Build algorithm, the degrees of freedom can be internal or external. Changes in external degrees of freedom transform the entire geometry of a ligand with respect to the central metal ion and/or to one another, whereas changes in internal degrees of freedom transform different parts of the geometry of a given ligand with respect to itself.

In the Complex Build algorithm, the only internal degrees of freedom considered are torsions, more specifically only those around proper dihedrals and not within a cycle, that we call free torsions. The set $\Phi$ of all unique free torsions can be computed using Algorithm 2. 
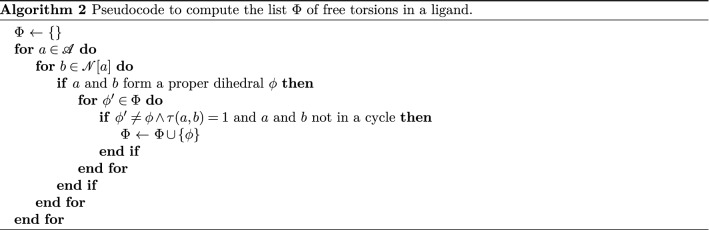


If we ignore the axis of a free torsion $\phi$, we split ${\mathscr {A}}$ into two sets: set ${\mathscr {A}}_1$ and set ${\mathscr {A}}_2$. Set ${\mathscr {A}}_1$ is the set of all atoms that remain connected to internal atom $i_1$, and likewise for ${\mathscr {A}}_2$. In order to obtain the relative change in position that is expected for the application of a free torsion, we must apply a rotation matrix to only one of these two atom sets. We then choose to do it on the smallest set of the two, denoted ${\mathscr {A}}_\phi$, with the other being denoted as ${\bar{{\mathscr {A}}}}_\phi$. These two sets can be computed using Algorithm 3. 
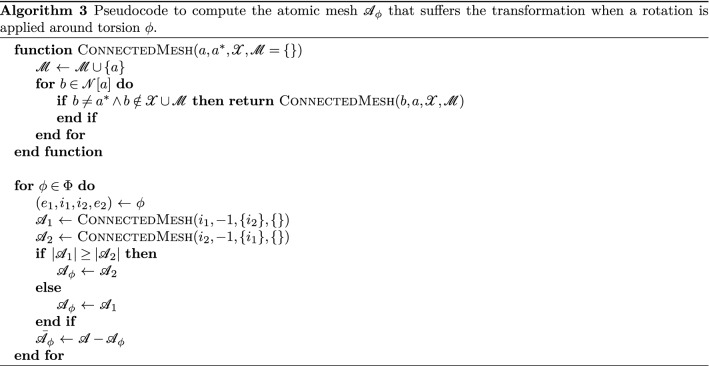


Given a torsion $\Phi$, the unit vector along its axis can be computed according to:

10 $$\begin{aligned} {\mathbf {u}}_\phi = \frac{{\mathbf {X}}[i_1]-{\mathbf {X}}[i_2]}{|{\mathbf {X}}[i_1]-{\mathbf {X}}[i_2]|} \end{aligned}$$

Vector ${\mathbf {u}}_\phi$ can be used to describe a rotation around $\phi$ by $\varphi$ degrees by the application of a matrix ${\mathbf {R}}_\phi (\varphi )$ to the position vectors of the atoms in ${\mathscr {A}}_\phi$. This matrix can be constructed incrementally by combining a series of intermediate transformations. We confine the axis unit vector ${\mathbf {u}}_\phi$ to the xz plane; we then rotate the vector in 2D such that it aligns itself with the z axis. Following this transformation, we apply the rotation by $\varphi$ degrees around the z axis. Afterwards, we revert the vector to its original orientation by undoing the alignment with the z-axis, the rotation in 2D and the projection to the xz plane. We now define ${\mathbf {u}}_\phi = (s,t,w)$, $d=\sqrt{s^2+t^2}$, $\mu = s/d$ and $\nu = t/d$. The first step is projecting the unit vector ${\mathbf {u}}_\phi$ onto the xz plane. This operation is represented by matrix ${\mathbf {T}}_{\text {xz}}$, which can be written as follows:

11 $$\begin{aligned} {\mathbf {T}}_{\text {xz}} = \begin{bmatrix} \mu &{} \nu &{} 0 \\ -\nu &{} \mu &{} 0 \\ 0 &{} 0 &{} 1 \\ \end{bmatrix} \end{aligned}$$

Following this operation, the unit vector ${\mathbf {u}}_\phi$ must be aligned with the z axis. This alignment is represented by matrix ${\mathbf {T}}_{\text {z}}$, written as:

12 $$\begin{aligned} {\mathbf {T}}_{\text {z}} = \begin{bmatrix} w &{} 0 &{} -d \\ 0 &{} 1 &{} 0 \\ d &{} 0 &{} w \\ \end{bmatrix} \end{aligned}$$

Lastly, the rotation around the new dihedral axis (which coincides with the z axis) is given by a matrix that represents a rotation around the z axis by $\varphi$ degrees, denoted by ${\mathbf {R}}_{\text {z}}(\varphi )$ and defined as:

13 $$\begin{aligned} {\mathbf {R}}_{\text {z}}(\varphi ) = \begin{bmatrix} \cos (\varphi ) &{} -\sin (\varphi ) &{} 0 \\ \sin (\varphi ) &{} \cos (\varphi ) &{} 0 \\ 0 &{} 0 &{} 1 \\ \end{bmatrix} \end{aligned}$$

The entire rotation around the dihedral $\phi$ by $\varphi$ degrees, denoted by the transformation matrix ${\mathbf {R}}_\phi (\varphi )$, can be summarized as the dot product of all of those operations and their inverses as shown in Eq. ( 14).

14 $$\begin{aligned} {\mathbf {R}}_\phi (\varphi ) = {\mathbf {T}}_{\text {xz}}^{{\mathsf {T}}}\cdot {\mathbf {T}}_{\text {z}}^{{\mathsf {T}}}\cdot {\mathbf {R}}_{\text {z}}(\varphi ) \cdot {\mathbf {T}}_{\text {z}} \cdot {\mathbf {T}}_{\text {xz}} \end{aligned}$$

Given such a matrix, the geometry matrix of the complex is updated as ${\mathbf {X}}[{\mathscr {A}}_\phi ] = {\mathbf {R}}_\phi (\varphi )\cdot{\mathbf {X}}[{\mathscr {A}}_\phi ]$, where ${\mathbf {X}}[{\mathscr {A}}_\phi ]$ represents the rows of ${\mathbf {X}}$ corresponding to the atoms in ${\mathscr {A}}_\phi$ that are transformed by the matrix ${\mathbf {R}}_\phi (\varphi )$.

External degrees of freedom are arbitrarily subdivided into two sets: orientational and positional. Orientational degrees of freedom are those related to the relative orientations between the ligands and the metallic center, but they generally do not change the relative positioning between the metallic center and the ligand’s geometric center, whereas positional degrees of freedom do.

**Figure 2:**
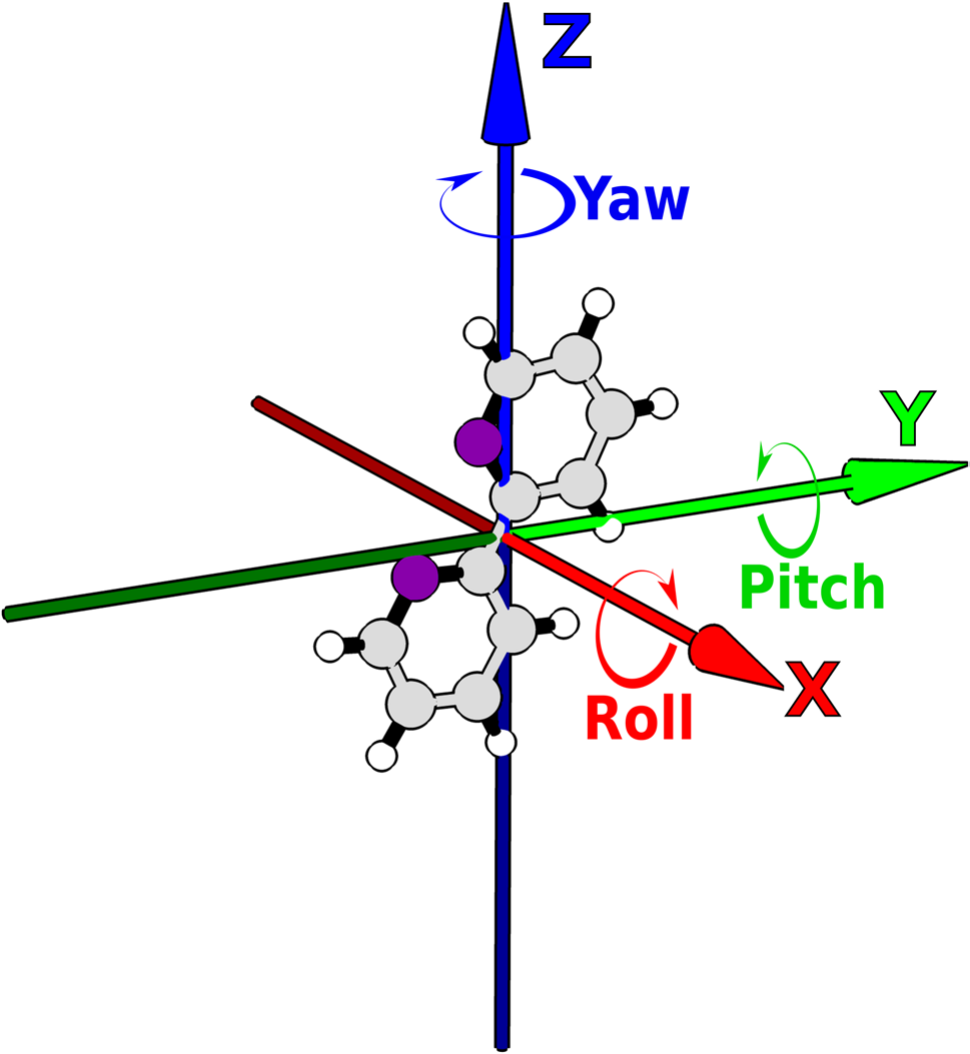
Euler angles centered at the ligand, here shown to be a 2,2-bipyridine. Nitrogens are colored purple so as not to be confused with the blue Z axis.

Orientational degrees of freedom are divided into two categories: single degree of freedom angles and Euler angles. Euler angles (Fig. 2) represent rotations around the Cartesian axes with the origin of this local coordinate system at the barycenter of the ligand. They represent the free change in orientation of the ligand around its own geometric center.

**Figure 3:**
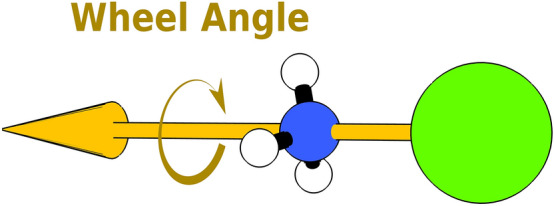
Examples of wheel angle for a monodentate ligand in a complex.

We now define two types of single degree of freedom angles: wheel angles and hinge angles. Wheel angles are defined for monodentate ligands as the angle of rotation around the axis that passes through the tooth of the ligand and the metallic center (Fig. 3).

**Figure 4:**
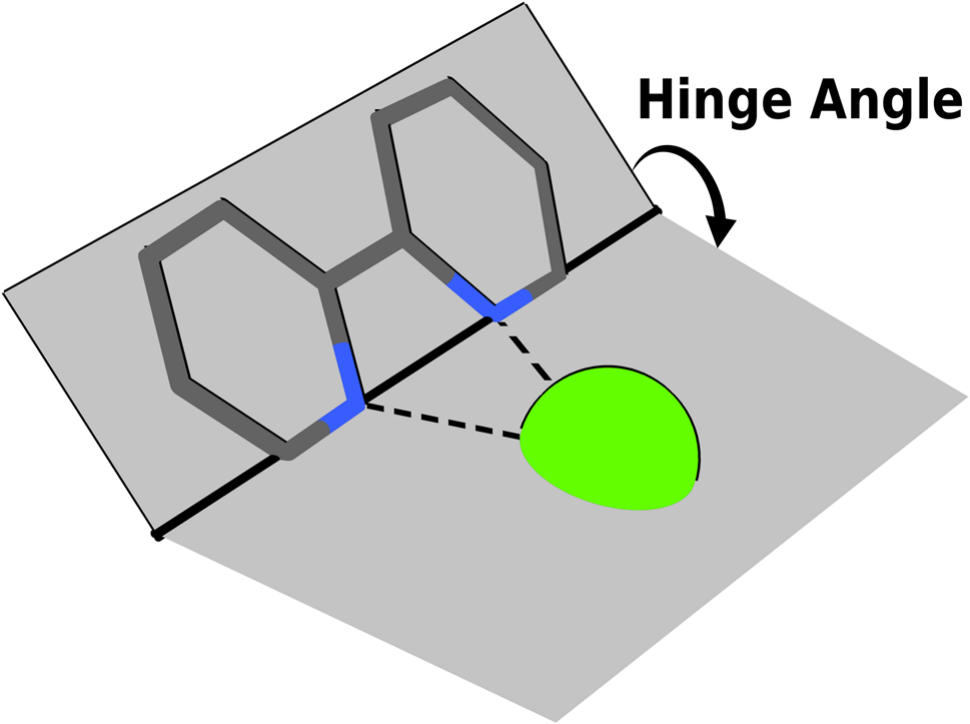
Sample illustration of a hinge angle for a bidentate ligand in a complex.

Hinge angles are defined for bidentate ligands as their angle of rotation around the axis that passes through both of its teeth (Fig. 4) intuitively similar to the angle defined by both planes of an open notebook. The wheel or hinge angle of a ligand is therefore a single degree of freedom intended to allow sufficient flexibility to the orientation of a ligand in a fixed-shape complex. Variations in wheel or hinge angles only represent reasonable motions if the teeth of the ligand are already suitably positioned towards the metallic center in fixed-shape complexes, as will be explained further in the next section. Alternatively, the flexibility provided by the single degree of freedom wheel and hinge angles, would already be contained in the Euler angles’ three degrees of freedom.

**Figure 5:**
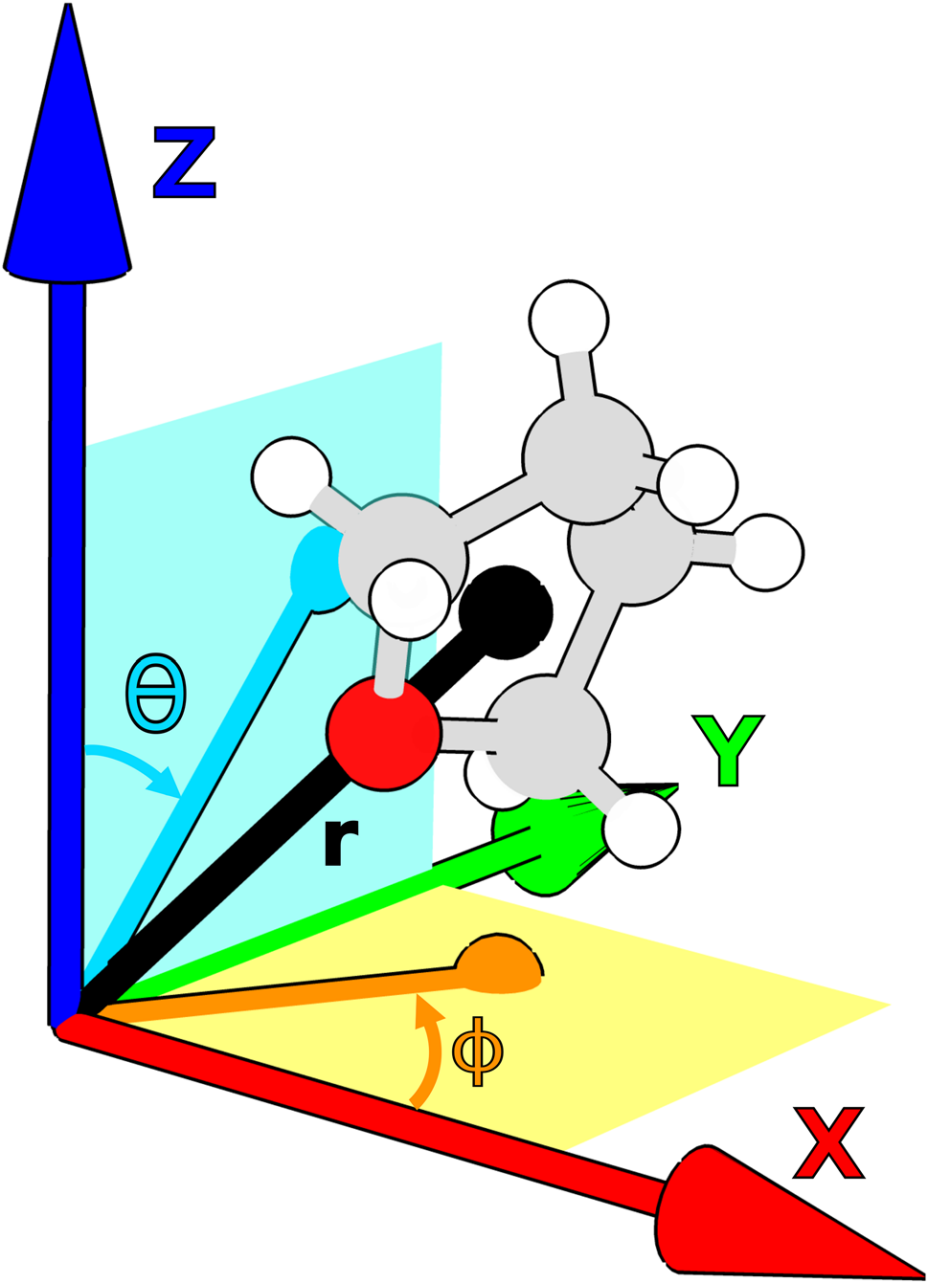
Spherical coordinates representing the position of a ligand in the coordination space. The barycenter of the ligand is represented as a black sphere, and its position vector as the line extending from the origin to the sphere. Position in the coordination ray is represented by the radial coordinate *r*, defined as the distance from the barycenter to the origin. The inclination $\theta$ is defined as the angle between the Z axis and the projection of the barycenter position vector into the ZY plane, shown in dark cyan. The azimuth $\phi$ is defined as the angle between the projection of the barycenter position vector in the XY plane and the X axis.

Positional degrees of freedom are also divided into two categories: position in the coordination ray and position in the coordination space. The coordination ray of a ligand is the half-line that originates in the metallic center and crosses the barycenter of the ligand. The distance from the metal to the ligand is its position in the coordination ray. Varying this position means sliding the ligand along the ray. The coordination space is the entire continuous space available to the ligands around the metal center, represented by a set of 3D spherical coordinates shown in Fig. 5. Thus, varying the position in the coordination space means translating the ligand in 3D space by the metallic center.

Because the position in coordination space also includes the radial spherical coordinate, any arbitrary variation in it may produce changes in the position of the ligand along its coordination ray. This creates conflicts between motion along the coordination ray and motion in the coordination space. For this reason, in the context of the Complex Build algorithm, both categories of motions cannot be varied simultaneously.

### The objective function

The purpose of the Complex Build algorithm is to provide a starting geometry for further molecular modeling calculations. In general, molecular modeling softwares are better at packing looser geometries in the correct way, rather than untangling any unfavorably interlaced and knotted geometry trapped in a local minimum of an intricate potential energy surface. Therefore, the Complex Build algorithm is being designed to ideally output lanthanoid complex geometries that could serve as starting geometries for molecular modeling softwares that, upon optimization by more sophisticated molecular mechanics or quantum chemical model chemistries, will ideally converge to optimized geometries without imaginary vibrational frequencies, indicative of true minima, hopefully good candidates to the global minimum on the potential energy surface.

To arrive at these desired starting geometries, the Complex Build algorithm first attempts to separate, as much as possible, all ligands from one another. This is accomplished by a fully repulsive potential, of the form $+1$/r, weighted according to each of the atom-atom interaction types. Atoms are classified into two groups: of hydrogens and of non-hydrogen atoms. Interactions between two non-hydrogen atoms are weighted 4, those between non-hydrogen and hydrogen atoms are weighted 2 and those between hydrogen atoms are weighted 1.

Of course, alone, this repulsive potential would end up infinitely scattering the ligands. To prevent that, a second potential, of the form of a potential well, must be superimposed to this repulsive one with the purpose of maintaining the coordinate bond distances within a reasonable range. Added, these two potentials are a measure of how closely packed or even how entangled a structure is. Crowding is the name we gave to this combined potential, which must be minimized in a geometry optimization procedure. We represent crowding by the greek letter $\Xi$. We chose this name for our potential, in an analogy to crowding in dentistry, which implies a condition in which the teeth are crowded in the dental arch, assuming altered positions, as by overlapping and twisting. Naturally, crowding ignores favourable interactions that might eventually occur between ligands, leading to constructed geometries that are generally much less “packed” than would be experimentally expected. Yes, these structures are not expected to be comparable with the experimental ones. The whole Complex Build algorithm has been designed so that they can be first-rate starting geometries for further, and much more sophisticated, molecular modeling calculations, which will then take into account all details of the fine interactions that were left out.

Indeed, in the Complex Build algorithm, crowding $\Xi$ is a sum of two quantities: the first is the steric congestion (*S*) which represents how close to one another each ligand atom is; and the second is the coordination warp (*W*), which encapsulates how deformed is the coordination polyhedron of the complex. Minimization of crowding with respect to the degrees of freedom of the ligands is the process of obtaining a solution to the problem.

The steric congestion is described as the sum of the inverse of the distances between each and every unique pair of atoms, as indicated by Eq. (15).

15 $$\begin{aligned} S = \sum _{n}^{|{\mathscr {A}}|} \sum _{m<n}^{|{\mathscr {A}}|} \frac{1}{|{\mathbf {X}}[n]-{\mathbf {X}}[m]|} \end{aligned}$$

Congestion is lowest when all the atoms are as far apart from one another as possible. How far they can get is limited by the degrees of freedom accessible to each ligand.

Numerical experiments have shown us that treating hydrogen atoms as equally repulsive as the rest of the atoms in the ligands often introduces excessive crowding that may lead to unnecessarily strained structures. We found out that this happens because hydrogen atoms are by far the most numerous and, since they only form a single bond, are more likely to be found in the extremities of the ligands, where they contribute a great deal to the crowding related to inter-ligand repulsion, as opposed to intra-ligand tension. Also, of all atoms that are likely to be present in an organic molecule, hydrogen atoms are, by far, the smallest.

For these reasons, for the purposes of computing the steric congestion, the atom set is partitioned into two: a subset of all hydrogen atoms, denoted ${\mathscr {A}}_H$, and another containing the remaining non-hydrogen atoms, denoted ${\mathscr {A}}_J$. Thus the total steric congestion *S* is given as a sum $S=S_{HH} + S_{HJ} + S_{JJ}$ in which $S_{HH}$, $S_{HJ}$ and $S_{JJ}$ are given by Eqs. (16a)–(16c), respectively.

16a $$\begin{aligned} S_{HH}&= \sum _{n}^{|{\mathscr {A}}_H|} \sum _{m<n}^{|{\mathscr {A}}_H|} \frac{1}{|{\mathbf {X}}[n]-{\mathbf {X}}[m]|} \end{aligned}$$

16b $$\begin{aligned} S_{HJ}&= 2 \sum _{n}^{|{\mathscr {A}}_H|} \sum _{m \le n}^{|{\mathscr {A}}_J|} \frac{1}{|{\mathbf {X}}[n] - {\mathbf {X}}[m]|} \end{aligned}$$

16c $$\begin{aligned} S_{JJ}&= 4 \sum _{n}^{|{\mathscr {A}}_J|} \sum _{m<n}^{|{\mathscr {A}}_J|} \frac{1}{|{\mathbf {X}}[n]-{\mathbf {X}}[m]|} \end{aligned}$$

We now turn to define the second potential: the coordination warp W. Depending on whether or not the complex has a coordination polyhedron with a predefined shape to it, the coordination warp will be defined by either Eq. (17a) or Eq. (17b)

17a $$\begin{aligned} W&= \sum _{n}^{|\Pi |} (|{\mathbf {X}}[n] - {\mathbf {Z}}[n]|)^2 \end{aligned}$$

17b $$\begin{aligned} W&= \sum _{n}^{|\Pi |} (|{\mathbf {X}}[n]| - r_0[n])^2 \end{aligned}$$

where ${\mathbf {X}}[n]$ denotes the position vector of the *n*-th tooth in the complex and ${\mathbf {Z}}[n]$ the position vector of the *n*th vertex in a reference shape geometry matrix ${\mathbf {Z}}$. Equation (17a) is thus the coordination warp when the complex has a predefined shape, encoded in matrix ${\mathbf {Z}}$, and is lowest when the atoms of the coordination polyhedron align more perfectly with the reference geometry indicated in ${\mathbf {Z}}$. On the other hand, the term $r_0[n]$ in Eq. (17b) refers to the target coordinate bond length between the *n*-th tooth in the coordination polyhedron and the central metal ion. The warp described by Eq. (17b) is lowest when the bond lengths are perfectly equal to their target values $r_0$. Accordingly, in Eq. (17b), the angular disposition of the ligands around the coordination sphere is free and controlled mainly by the steric congestion *S*.

Crowding is therefore defined as the sum of the steric congestion and the coordination warp. Because the absolute value of *S* is much larger than that of *W* (on account of being a sum over a much larger set of terms), a parameter is required to equilibrate the value of warp so that it becomes non-negligible, as shown in Eq. (18).

18 $$\begin{aligned} \Xi = S + \alpha W \end{aligned}$$

Through extensive numerical experimentations, we determined that a reasonable value for $\alpha$ that works for a wide range of sizes and types of complexes is the one shown in Eq. (19).

19 $$\begin{aligned} \alpha = 100 \frac{|{\mathscr {A}}|}{\sqrt{|\Pi |}} {\AA}^{-3} \end{aligned}$$

### Target coordinate bond lengths

To build the starting structures to be preliminarily optimized by the Complex Build algorithm, we need appropriate values for the coordinate bond lengths, since they are required to compute the coordination warp, with or without shape constraints.

In practice, only a subset of elements have atoms that are capable of forming stable coordinate bonds with metallic ions. Accordingly, we tried to obtain reasonable values for the bond lengths of these naturally-occurring coordinating atoms from a comprehensive set of complexes. For this first version of the Complex Build algorithm, we decided to focus on lanthanoid complexes.

Our dataset was a subset of the one employed in the parametrization process of the Sparkle/RM1 model^20,21^. It includes a total of 815 structures of lanthanoid complexes with a single trivalent cation as the metallic center, carefully chosen to be representative of the larger set of complexes for each metal. We then computed the median and the median absolute deviation (MAD) values of the coordinate bond lengths for all the types of coordinating atoms that appeared in this set of structures for trivalent lanthanoid cations.

The median of a distribution is the value such that half the distribution lies above it and half below. So if *x* is a median coordinate length and ${\text {MAD}}$ is its median absolute deviation, then the value $x+{\text {MAD}}$ was chosen to be our target coordinate bond length $r_0$ defined for each lanthanoid under the assumption that a more loose coordination can be more properly compressed later by a molecular modeling software, whereas a tighter coordination might generate a more densely packed structure that may find itself trapped in poor local minima upon further optimization with a molecular modeling software. The results are summarized in Table 2.

**Table 2:** Target coordinate bond lengths in picometers (pm) between the lanthanoid ions and the types of coordinating atoms found in our dataset.

	O	N	S	F	Cl	Br	I	P
La	*269*	*280*	297	260	274	290	331	304
Ce	*263*	*270*	296	259	273	289	330	303
Pr	*262*	*275*	293	256	270	286	327	300
Nd	*260*	*268*	*296*	255	*287*	*293*	326	299
Pm	*254*	*273*	*314*	*254*	*264*	283	325	298
Sm	*253*	*264*	*293*	253	*283*	*305*	324	297
Eu	251	265	*289*	252	*266*	*282*	322	296
Gd	*247*	*270*	288	251	*264*	*282*	322	295
Tb	*248*	*263*	287	249	*273*	280	320	293
Dy	*245*	*266*	*290*	248	*261*	*287*	*319*	*292*
Ho	*243*	*260*	*300*	247	*263*	277	318	291
Er	*245*	*251*	*285*	246	*272*	*285*	317	290
Tm	*242*	*257*	282	245	259	275	316	289
Yb	*241*	*260*	281	244	258	274	315	288
Lu	*241*	*252*	280	243	257	273	314	287

Values in italic were computed directly from the database, and the remaining values had to be interpolated from other lanthanoids.

Not every combination of lanthanoid-coordinating atom was represented in the dataset. To counter that, we devised a method to estimate these target bond distances from the data obtained for the other lanthanoids.

Let the radius of an ion *X* be denoted $\varrho [X]$ and the target coordinate bond length between an ion $M^{+m}$ and a coordinating atom *t* in the database be denoted as $r_0[M^{+m},t]$. Now suppose that we have a different ion $N^{+n}$ for which $r_0[N^{+n},t]$ is undefined because no instances of a bond of this type appear in the dataset. The value for this missing bond length is estimated using the relationship shown in Eq. (20)

20 $$\begin{aligned} r_0[N^{+n},t] = r_0[M^{+m},t] - \varrho [M^{+m}] + \varrho [N^{+n}] \end{aligned}$$

in this relation, $N^{+n}$ is the result-ion whereas $M^{+m}$ is the argument-ion. For the best use of this method, the argument-ion should have a large representation of bonds of the type $M^{+m} \cdots t$ in the dataset such that we can be confident about the values we obtained from the set. In most cases, we used ${\text {Eu}}^{+3}$ as the argument-ion, simply because complexes with it were much more numerous in our dataset than with any other lanthanoid trications.

However, not every type of coordinating atom in the database had bonds with europium. The bonds between trivalent europium and fluorine, iodine and phosphorus were notably missing. For these types of coordinating atoms we used as argument-ions, dysprosium for phosphorus and iodine; and promethium for fluorine. These lanthanoids were chosen as argument-ions because they had more bonds with these coordinating atoms than any other lanthanoid.

This relationship works for lanthanoid ions because their valence orbitals are more internal to the atom than their core orbitals. The electrons of the outer orbitals do not deform the external electron density of the lanthanoid, which gives it a somewhat smooth spherical shape and makes the interaction between ion and ligand essentially electrostatic. Because the shape of the ionic electron density is preserved across the entire lanthanoid series, the only relevant difference in their coordinate bond lengths is their ionic radius, which decreases from La to Lu due to the lanthanide contraction. The effective ionic radii we used^22^ as $\varrho$ are shown in Table 3.

**Table 3:** Effective ionic radii in picometers (pm) of lanthanoid ions, of both oxidation states, used in the method for the interpolation of coordinate bond lengths.

Lanthanoid	Oxidation state	
	$+2$	$+3$
La	*146*	103.2
Ce	*140*	101.0
Pr	*135*	99.0
Nd	129	98.3
Pm	*125*	97.0
Sm	122	95.8
Eu	117	94.7
Gd	*113*	93.5
Tb	*110*	92.3
Dy	107	91.2
Ho	*106*	90.1
Er	*104*	98.0
Tm	103	88.0
Yb	102	86.8
Lu	*101*	86.1

Values in italic were obtained from the quadratic function in Eq. (21).

The table does not include many of the divalent lanthanoid ionic radii, but the fit of a quadratic function to the values that we do have, yields the quadratic function shown in Eq. (21)

21 $$\begin{aligned} \varrho [{\text {Ln}}^{2+}] = 0.2097 Z^2 - 30.043 Z + 1177.2 \end{aligned}$$

where $\varrho [{\text {Ln}}^{2+}]$ is the target coordinate bond length of the divalent lanthanoid cation and Z is its atomic number. This expression has an $R^2 = 0.995$ across the entire series of lanthanoids. Table 3 was then completed for all the lanthanoid dications using this function.

Using an additional 14 structures of divalent europium complexes along with the predicted ionic radii for divalent cations found in Table 3, and the relation in Eq. (20), we used divalent europium as the argument-ion to estimate target bond lengths for all divalent lanthanoid cations, for N, O and P, that are the coordinating atoms found in the divalent europium set. Table 4 shows these target coordinate bond lengths for the divalent cations.

**Table 4:** The target coordinate bond lengths (pm) between divalent lanthanoid ions and the types of coordinating atoms found in our dataset.

	O	N	P
La	295	304	342
Ce	289	298	336
Pr	284	293	331
Nd	278	287	325
Pm	274	283	321
Sm	271	280	318
Eu	*266*	*275*	*313*
Gd	262	271	309
Tb	259	268	306
Dy	256	265	303
Ho	255	264	302
Er	253	262	300
Tm	252	261	299
Yb	251	260	298
Lu	250	259	297

Values in Italic were computed directly from the data, whereas the remaining values had to be extrapolated by using Europium as the argument metal.

### Shapes of coordination polyhedra

A shape ${\mathscr {Q}}$ is represented in the Complex Build algorithm as a tuple, such that ${\mathscr {Q\!}}\,= \{{\mathscr {Z}},{\mathbf {Z}},{\mathscr {S}},c,T\}$ where ${\mathscr {Z}}$ is a set of vertices in three-dimensional space and ${\mathbf {Z}}$ is the geometry matrix, related with ${\mathscr {Z}}$ in the same way that the set of atoms ${\mathscr {A}}$ of an atomic and molecular structure is related to its geometry matrix ${\mathbf {X}}$. Furthermore, ${\mathscr {Z}}$ is partitioned, and ${\mathscr {S}}$ is the set of these partitions of ${\mathscr {Z}}$, called sites, as defined by Eq. (22).

22 $$\begin{aligned} \forall {\mathscr {s}}_1, {\mathscr {s}}_2 \in {\mathscr {S}}{\mathscr {s}}_1 \cap {\mathscr {s}}_2 = \varnothing \wedge {\mathscr {s}}_1, {\mathscr {s}}_2 \in {\mathscr {Z}}\end{aligned}$$

Moreover, we have that $\bigcup _{{\mathscr {s}}\in {\mathscr {S}}} {\mathscr {s}}= {\mathscr {Z}}$, meaning that every vertex of the shape will be part of some site, even if it is just a site composed only of itself.

The partition of the shape into sites must follow the set of ligands in a complex. For every ligand in the complex, there has to be a site with a size equal to the denticity of that ligand. Or, inversely, given a specific set of partitions, the ligands must be chosen so as to match them completely, without leaving a single site vacant. Thus $\forall {\mathscr{l}}\in {\mathscr{l}}\exists {\mathscr {s}}\in {\mathscr {S}}| |{\mathscr {s}}| = |{\mathscr{t}}\subset {\mathscr{l}}|$.

Like teeth, vertices also receive an identification index called a “color” $c:{\mathscr {Z}}\rightarrow {\mathbb {N}}$ that tells which abstract ligand can get attached to each site. For example, given a shape with four vertices, all of the same color, all of its vertices must be occupied by four teeth of the same color as each other. So either the four vertices are all part of the same site, to which a tetradentate with chemically equal teeth is bound; or they are split into two sites of two vertices, each one occupied by identical bidentate ligands with chemically identical teeth; or each is part of a single vertex site, each occupied by a molecule of the same monodentate ligand. The point is that the color tells us the chemical identity of the teeth allowed at each site, in comparison with the other teeth in the ligand set.

Given a shape with colored vertices, and a complex with colored teeth, we must associate each ligand to a single site. In that context, we denote as ${\mathscr {S}}^n$ the subset of ${\mathscr {S}}$ containing only the sites with *n* elements. Likewise, ${\mathscr{L}}^n$ is the subset of ${\mathscr{L}}$ containing only ligands of denticity *n*. In a similar way as a ligand, a site ${\mathscr {s\!}}\in {\mathscr {S}}^n$ also has a type that can be accessed by the function $T:{\mathscr {S}}^n \rightarrow {\mathbb {N}}^n$. $T({\mathscr {s\!}})$ is a tuple in which every element is of the color of one of its composing vertices.

We also define set ${\bar{{\mathscr {S}}}}$ of sites that are already bound. With these, we can associate each ligand to a single site according to Algorithm 4 below: 
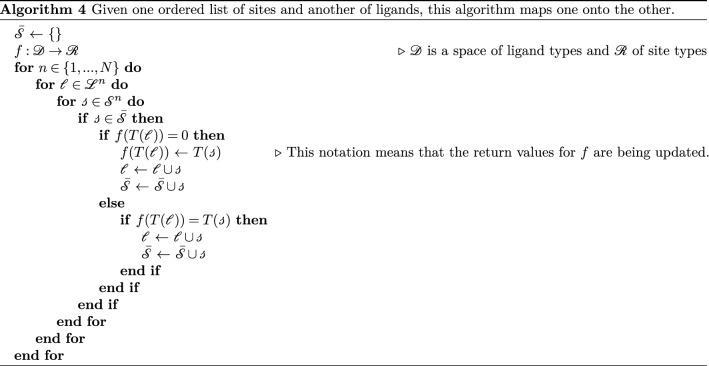


After running through this algorithm, every ligand ${\mathscr{l}}$ of a complex will be united with the set ${\mathscr {s}}$ of the site to which it is bound. A ligand of this type ${\mathscr{l}}\cup {\mathscr {s\!}}$ is said to be a bound ligand. For a sequence of bound ligands $\{{\mathscr{l}}_n\}$ , their respective sites can be denoted by the notation ${\mathscr {s}}_n$.

One additional side effect of this algorithm is the determination of the relationship between the types of sites and the types of ligands through function *f*. Once this association is known, we can order the rows of the geometry matrix ${\mathbf {Z}}$ to line up, one to one, with the rows of the coordination polyhedron ${\mathbf {X}}[\Pi ]$.

Once the rows are matched between the two matrices, we must then rescale the “coordinate lengths” of each vertex, which is the equivalent of rescaling their position vectors. Given a pair of associated vertex and tooth (*v*, *t*), the shape geometry is updated as:

23 $$\begin{aligned} {\mathbf {Z}}[v] = \begin{bmatrix} r_0[\text {Me},t] &{} 0 &{} 0 \\ 0 &{} r_0[\text {Me},t] &{} 0 \\ 0 &{} 0 &{} r_0[\text {Me},t] \cdot {\mathbf {Z}}[v] \end{bmatrix} \end{aligned}$$

where $r_0[\text {Me},t]$ is the target coordinate bond length between the metallic center of the complex and the particular tooth type associated with the vertex. After this update, the shape and the set of ligands are entirely lined up and equivalent.

When building a complex with a fixed shape, the Complex Build algorithm aims to reduce the difference ${\mathbf {X}}[\Pi ]-{\mathbf {Z}}$ as much as it can to preserve the particular spatial arrangement and coordinate bond lengths of the complex during the final optimization algorithm to find the final solution geometry.

In order to build a complex with fixed shape, it is necessary to choose a shape and to associate each ligand in ${\mathscr{L}}$ with a set of vertices, called a site, of the shape. The complete set of shapes obtained for the lanthanoid complexes in our database has been presented in a different work^16^.

### Geometry initialization and optimization

At the center of the Complex Build algorithm is the optimization of the geometry matrix of the complex with respect to its crowding. The solution matrix is the one which minimizes the crowding. Since the optimization process is local, it requires the input of some starting structure. The process of initializing the structure is different depending on whether the complex has a fixed shape or not.

When a complex does not have a defined shape, we spread the ligands out in the coordination sphere as evenly as possible. The problem of spreading points evenly on the surface of a sphere does not have a unique solution, largely because there are many different (and equally valid) criteria with regards to which the resulting point cloud should be optimal. However, there are many approximate solutions that will provide sufficiently spread out point clouds for our purpose. One such solution starts with the Fibonacci lattice on a sphere.

**Figure 6:**
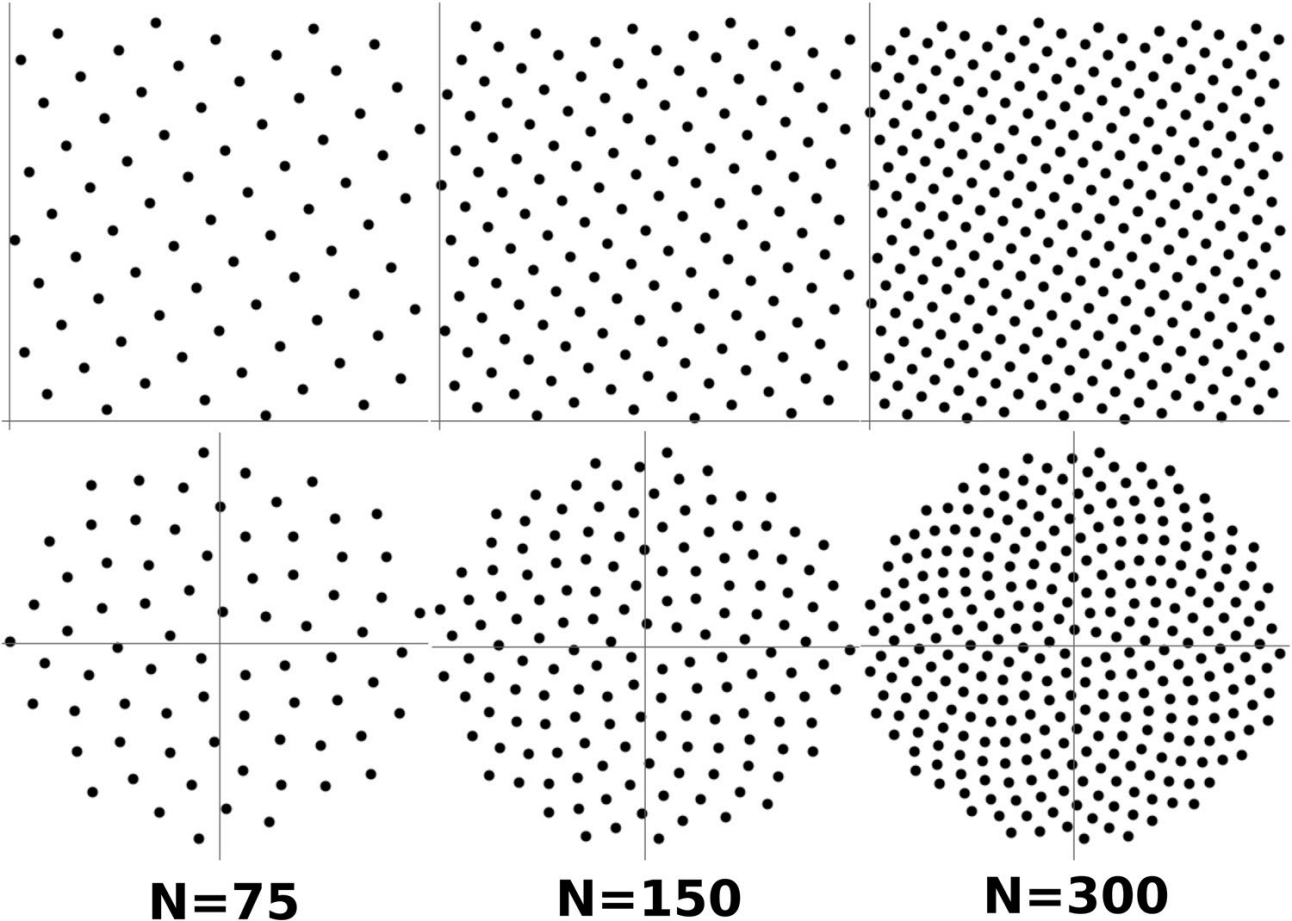
Top row shows the Fibonacci lattice in the unit rectangle for n=75, 150 and 300 points respectively, from left to right. On the bottom row we see the Fibonacci spirals, the mapping of the lattice into polar coordinates in the unit circle.

The Fibonacci lattice for *N* points in the unit square is given by Eq. (24), shown below,

24 $$\begin{aligned} t_n = (x_n,y_n) = \left[ \left( \frac{n}{{\phi }}\right) \%1, \frac{n}{N} \right] \forall 0 \le n \le N \end{aligned}$$

where the notation $(\cdot )\%1$ means the fractional part of the argument and ${\phi }$ in the particular context of Eq. (24), means the Golden Ratio. Mapping this lattice to the unit circle gives a reasonable distribution for evenly spaced points, particularly for large values of *N*. This mapping can be done via the area-preserving transformation shown in Eq. (25)

25 $$\begin{aligned} (x,y) \rightarrow (\theta ,r) : (2 \pi x, \sqrt{y}) \end{aligned}$$

where $\theta$ and *r* are the polar coordinates and *x* and *y* are the Cartesian coordinates. This mapping gives rise to a distribution that can be fitted using Fibonacci spirals. The lattices in Cartesian and polar coordinates can be visually compared in Fig. 6 below, for different numbers of points.

Likewise, the Fibonacci lattice can also be mapped from the unit square onto the surface of the unit sphere, and from there to the 3D cartesian space using the relations in Eqs. (26a) and (26b), respectively.

26a $$\begin{aligned} (x,y)&\rightarrow (\theta ,\phi ):(2 \pi x, \arccos (1-2y)) \end{aligned}$$

26b $$\begin{aligned} (\theta ,\phi )&\rightarrow (x,y,z):(\cos \phi \cos \theta , \sin \phi \sin \theta , \cos \theta ) \end{aligned}$$

**Figure 7:**
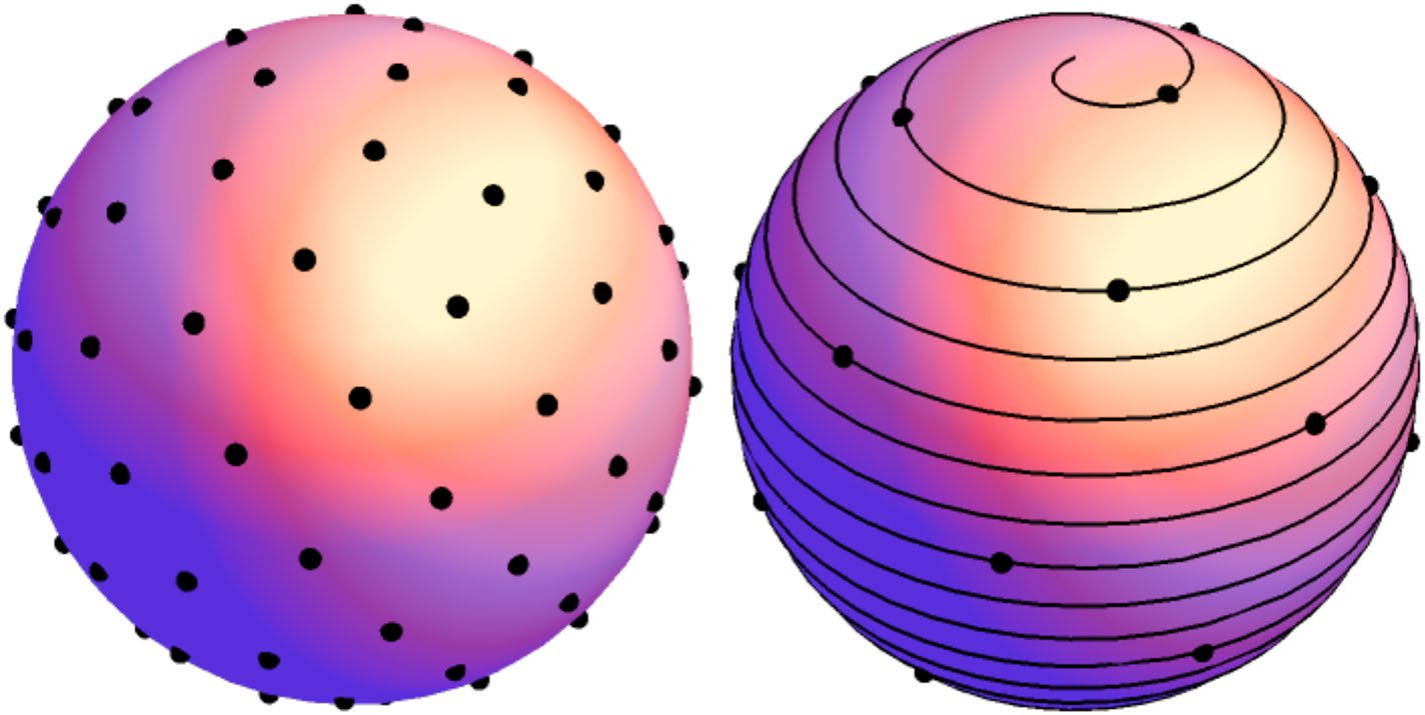
Example of a Fibonacci lattice (left) on the surface of a sphere and one of the Fibonacci spirals that generated it (right). The dots on the spiral are the ones that are included in the lattice on the left.

In the context of the 3D spherical polar coordinates, $\theta \in [0,\pi ]$ is the latitude (or inclination) whereas $\phi \in [0,2\pi ]$ is the azimuth (or longitude). This gives a very evenly spread grid of points on the surface of the sphere that can also be assembled using Fibonacci spirals as shown in Fig. 7 below.

Once we have a set of points that form a Fibonacci lattice for $N=30$ points, we order this list such that the second point in the list is the farthest point in the lattice from the first, the third is the farthest point from the two that came previously and so on. The list is ordered in such a way that each point maximizes the distance to its predecessors. This can be done by submitting a list ${\mathscr {E}}$ of points computed from a spherical Fibonacci lattice as input to Algorithm 5. 
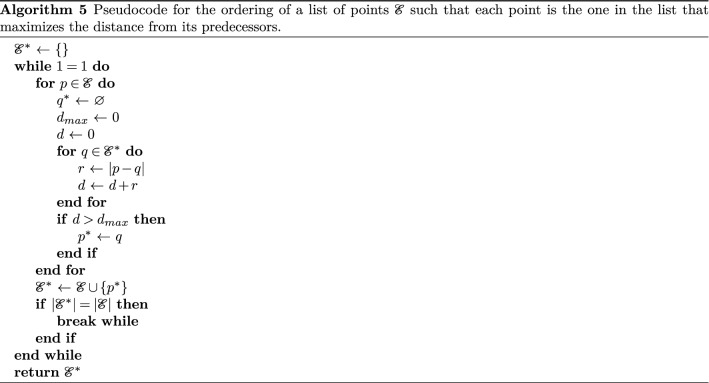


Once the ordered list ${\mathscr {E\!}}^*$ is obtained, its first $|{\mathscr{L}}|$ points are chosen as the starting positions of the ligands. This generates an evenly spread distribution of ligands, maximally spaced within the set of points produced by the lattice formula.

Complexes with a fixed shape utilize a different algorithm for initializing their starting geometries, since they have sites associated with them, and their teeth must be placed at the specific positions defined by their sites, or else the objective function will accuse a high crowding.

Simply placing the teeth of a ligand over its corresponding vertices in a site is not enough to guarantee its correct orientation relative to the coordination center. For instance, a bidentate ligand can be in any configuration accessible by rotation of its hinge angle without changing the positions of its teeth. From the point of view of a naive anchoring algorithm, all such configurations would, in principle, be valid initial positions. However, configurations that would place the bulk of the ligand over the metal are not realistic. We need to position the ligand in such a way that its teeth point towards the ion metal center, and its bulk is placed radially away from it. In order to get such specific orientation, we require reference points to be generated for all ligands and sites. For a given site ${\mathscr {s}}$, we denote ${\mathscr {s\!}}^*={\mathscr {s}}\cup \{\mathbf{p }^*\}$ the effective site, where $\mathbf{p }^*$ is the reference point of site ${\mathscr {s}}$. The reference point $\mathbf{p }^*$ is given by Eq. (27), shown below

27 $$\begin{aligned} \mathbf{p }^* = \begin{bmatrix} d &{} 0 &{} 0 \\ 0 &{} d &{} 0 \\ 0 &{} 0 &{} d \end{bmatrix} \cdot \mathbf{p } \end{aligned}$$

where $\mathbf{p }$ s the position vector of the centroid of its vertices, and *d* is a free parameter, set to 1.7. For a ligand, the reference point is given by the centroid of the position vectors of every atom in the neighborhood of its teeth, shown in Eq. (28).

28 $$\begin{aligned} \mathbf{p }^* = avg({\mathbf {X}}[{\mathscr {N}}[{\mathscr{t\!}}]) \end{aligned}$$

We can now define an effective teeth set ${\mathscr{t\!}}^* = {\mathscr{t\!}}\cup \{\mathbf{p }^*\}$. For each bound ligand, we thus have its effective teeth set ${\mathscr{t\!}}^*$ and its associated effective site ${\mathscr {s\!}}^*$. These two sets are then aligned with one another using the Kearsley algorithm^23^.

The application of the Kearsley algorithm generates the rotation matrix that superimposes both sets of points. This matrix is then used to rotate all the atoms of the ligand such that its teeth and reference point will coincide with the vertices and reference point of its site. The procedure is then repeated for each ligand until each of them has been initialized.

Once the geometry is initialized, optimization of the crowding function with respect to the accessible degrees of freedom may begin. Currently we use a trust region method^24^ together with a convergence threshold of 10^-4^Å^-1^ in the objective function.

### Coordination stereoisomerism

**Figure 8:**
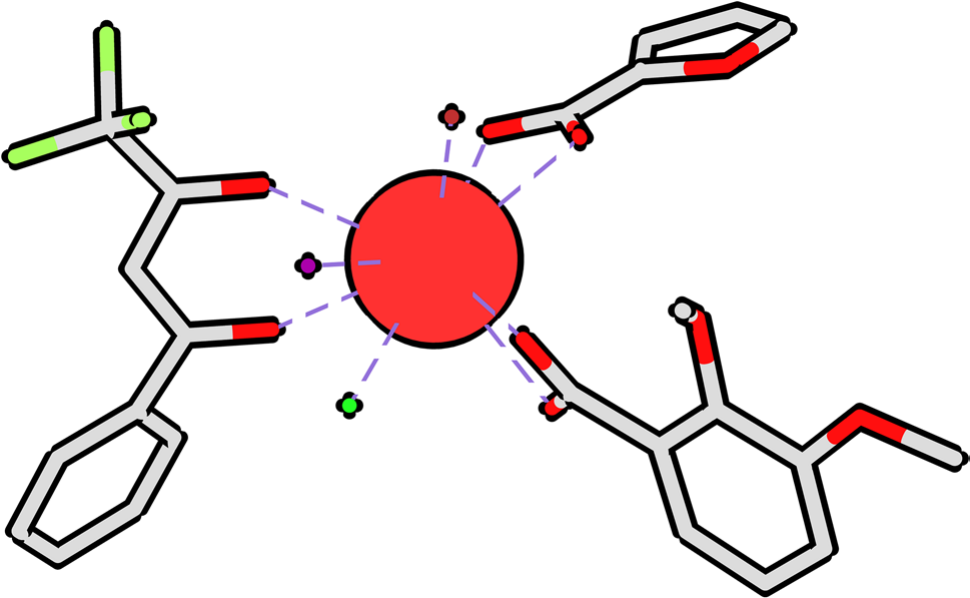
Example of a possible trivalent europium complex of generic formula Mabc(AB)(CD)(EF)^16^ of coordination number nine with 66,816 conceivable coordination stereoisomers. Coordinate bonds are represented as dotted lines.

Since coordination centers may be bound to a lot of different ligands, they have strong stereogenic potentialities and are capable of a much richer and intricate stereochemistry than the usual one of carbon. The scale of lanthanoid stereoisomerism is impressive. Even relatively simple complexes of coordination number nine show a staggering amount of over sixty thousand conceivable stereoisomers, as is the case of the one shown in Fig. 8.

This number is even more impressive in light of the fact that lanthanoids are capable of routinely forming complexes with coordination numbers as high as 11 or 12. Not every permutation in the rows of the shape matrix counts as a different, valid stereoisomer. Conversely, many permutations may indeed generate the same stereoisomer, only rotated to a different orientation. The process of exhaustively identifying which permutations correspond to distinct isomers was carried out and published by our group for a wide selection of shapes and every combination of mono and bidentate ligands available to these shapes up to coordination number 9^16^. The Complex Build algorithm is designed to be fully compatible with the entirety of that dataset and can employ the information therein to construct structures by using the present formalism regarding shapes, sites and permutations. Accordingly, each stereoisomer is encoded as a permutation on the rows of the ${\mathbf {Z}}$ matrix of the shape, and on the vertices that make up the sites of the shape. Since this is how the stereoisomers are represented in the Complex Build algorithm, it follows that a shape (and its geometry matrix ${\mathbf {Z}}$) is required to construct specific stereoisomers of a complex.

The Complex Build algorithm can incorporate the full range of potential stereochemistry accessible to complexes with mono or bidentate ligands and, therefore, to selectively generate starting structures for the molecular modeling of these species, allowing a more in-depth theoretical research on the stereochemistry of the complexes.

### Coordination chirality

Although the specific geometry of a metallic complex is a property of its underlying chemical topology and requires a full description of the relative positions and priorities of the various groups connected to the metal, the chirality of an object is not a concept that is exclusive of chemistry, and is a product of the symmetry of an object with respect to reflection, regardless of any other aspects of its internal structure.

Whereas a library of exhaustively obtained stereoisomers^16^ is used in the Complex Build algorithm to construct structures with controlled stereochemistry, the chirality of the coordination system of these structures is determined using a separate algorithm, applicable even to complexes constructed without a fixed shape.

The main problem in deciding whether a structure is chiral or not is that many atoms in the structure are equivalent to one another. For example, in a methane molecule, every hydrogen is equivalent. As such, when we try to superimpose methane with its own specular image, any of the hydrogens can be placed on top of any other for the two structures to be considered superimposable and equivalent. Indeed, if we fail to account for this chemical similarity between the various atoms or ligands bound to a center, then each atom would only be considered to be equivalent to itself, and the algorithm would erroneously classify methane as chiral.

In the Complex Build algorithm, the chemical similarity that allows for the matching of distinct atoms to one another is abstracted away through the use of precedence indices, similar to the ones used in the Cahn-Ingold-Prelog rules for tetrahedral stereoisomerism nomenclature. Each chemically distinct tooth of each type of ligand is given a different precedence order. As such, each atom in the coordination polyhedron will be given a precedence number that represents the entirety of its chemical environment. Because this methodology aims at determining the chirality of the coordination center only, the entire structure of the complex is not considered, just the coordination polyhedron and the precedence values associated to each of its vertices.

The exhaustive way of testing the chirality of a stereogenic center would be to try to superimpose the mirrored and unmirrored structures in accordance to every possible bijection between all the chemically equivalent atoms in the structure and their counterparts in the specular image. Once a bijection is decided between the atoms of the untransformed structure and those of the reflected structure, the two sets can be superimposed into one another using the Kearsley method^23^. The resulting structures can then be compared with each other using a measure of geometric dissimilarity (often the Root-Mean Square Deviation, RMSD) against a threshold to decide on their effective equality. Normally, the differences between values of similarity in successfully superimposed structures and in non-superimposable ones, are very distinct.

The problem with this approach is that, as the coordination number grows along with the number of chemically equivalent coordinating atoms, the amount of combinations between them grows very fast, making this a very impractical approach.

The problem is solved by the algorithm of Marques et al^23,25^. In this method, at each iteration the mirrored structure is rigidly rotated to a random orientation and, from this orientation, a specific bijection between the mirrored and the untransformed structures is computed. This computation relies on a matrix that determines how costly it would be to pair each atom of the mirrored structure with each atom in the untransformed structure. For our purposes, the cost for pairing atoms of same precedence is equal to the distance separating them, such that atoms would be preferentially paired with their closest equivalent in the mirrored structure. The cost of pairing atoms of different precedence value was set to $10^{10}$ times the separation distance, in order to ensure that atoms of different precedence number would never be paired together, no matter how close they were.

Once the cost matrix is decided, the bijection can be obtained using the linear sum assignment method^26^, often called the Hungarian method. With the bijection determined for the pair of structures, the Kearsley algorithm can be used to assess whether they are superimposable. The algorithm goes on like this, generating a random orientation of the specular image, computing the bijection between the atoms in each structure and testing for superposition at every iteration.

The coordination polyhedron will be chiral if, at every iteration, the RMSD between its original geometry and its mirror image stays above a certain threshold (that Marques sets at around 0.14 Å). If the RMSD measure crosses down that threshold at any iteration, the structure is said to be achiral. The more numerous the iterations, the more certain we can be that the chirality has been definitively determined. Based on the results of Marques et al, as well as our own numerical experimentation, we have decided that 10,000 iterations is more than enough to establish chirality.

## Results and discussion

We emphasize, once again, that the purpose of the Complex Build algorithm is not to attempt to predict experimental geometries, but rather to produce good starting geometries for subsequent molecular modeling calculations, focusing on the coordination polyhedron, more specifically on its stereochemical control. Human cognition does not easily grasp the rapid growth of complexity due to the combinatorial explosion of possibilities of stereoisomers in higher coordination metal compounds. Besides, the numerous degrees of conformational freedom of lanthanoid complexes would make the exploration of their entire conformational space essentially intractable via highly accurate and highly sophisticated quantum chemical methodologies. Therefore, scientists very commonly use either crystallographic geometries or a single structure they assembled, almost at random, as starting points for further molecular modeling calculations, all oblivious to the fact that such a structure is nothing short of just one out of several, sometimes dozens, or even thousands of other possible stereoisomers. Moreover, coordination chirality, so frequently present in complexes of higher coordination numbers, is another often overlooked property, rarely recognized as such^16^. Indeed, the Complex Build algorithm is being advanced here in this article to address all these issues.

The Complex Build algorithm does not include in its crowding formula any attractive interactions—it simply spreads the ligands out as much as is chemically feasible. We thus certainly expect some disagreements between a starting structure generated by the Complex Build algorithm, and the structure found experimentally in the crystal structure data, even when they correspond to the same stereoisomer. Likewise, we also expect some degree of disagreement between the crystal structure used as a starting geometry and its quantum-mechanically optimized one, since molecular modeling calculations are frequently carried out for isolated molecules, whereas the experimental compound, on the other hand, is usually confined to a solid lattice.

We thus regard, as a reasonable assessment of the quality of a starting geometry, a comparison between the final fully optimized geometries (by the very same quantum mechanical model) obtained from the following two starting geometries for a given complex: (1) a crystallographic structure; and (2) the Complex Build one with the same stereoisomer coordination stereochemistry as its crystallographic counterpart. Stereoisomerism must be equal in both structures to make sure that they correspond to the same compound.

Accordingly, we randomly selected a sample of complexes, one for each of the 14 lanthanoid trivalent ions with publicly available crystallographic data deposited in the Cambridge Structural Database (CSD)^27^ and whose stereochemistry had already been thoroughly identified in terms of their coordination polyhedra shapes and permutations, according to the methodology described by Silva et al.^16^. For this comparison, we are not concerned with the quantum chemical model per se, but rather with the performance of the starting geometries in leading to equivalent final molecular modeling optimized complex structures. Accordingly, we generated Complex Build starting geometries for these complexes with the same stereochemistry (and thus with the same point group, with all the coordination polyhedron symmetry elements unchanged) as their crystallographic experimental ones, and fully optimized both sets (Complex Build and crystallographic) with the same quantum chemical model: RM1 model for lanthanoids^28–32^, available in MOPAC 2016—our choice for this study since RM1 has been proven to lead to good coordination polyhedron geometries, comparable to those obtained by crystallography, in average within 0.06 Å of their correct values^33^, which is sufficient for our purposes.

The Complex Build algorithm purposefully eases the structure from tighter configurations in the interest of generating looser structures that are less likely to be trapped into sub-optimal local minima during the molecular modeling optimization and, therefore, are also less likely to lead to quantum chemically optimized geometries displaying imaginary vibrational frequencies. So much so, that we deem as a second measure of starting geometry quality, how few imaginary vibrational frequencies the corresponding quantum chemically optimized geometry displays.

In summary, good starting geometries for subsequent energy-based quantum mechanical full optimizations can be regarded as ones that will lead to true minima of low energies, ideally with no imaginary frequencies, and similar to the structures that would be otherwise obtained had the starting geometry be an experimental one. Results for the 14 complexes, chosen as a representative sample, one for each lanthanoid trication with the sole exception of the exceedingly rare and radioactive element promethium, are shown in Table 5. The complexes display several shapes and coordination numbers ranging from 6 to 9 in this sample.

**Table 5:** Fifteen representative lanthanoid metal complexes are listed, one for each element in the series, along with their generic formula, as well as the point group, PG, and isomer permutation of its coordination polyhedron crystallographic structure.

	CSD code	Generic formula	Shape	PG	Isomer permutation	$\Delta \Delta _f H$ (kcal/mol)
La	QAKWEE	[La](AA)4	TDD-8	$D_2$	[1 4 3 2 5 8 7 6]	$-2.4$
Ce	PUTQAW	[Ce](AB)4	SAPR-8	$C_2$	[1 4 3 2 8 7 5 6]	19.5
Pr	ZAXRUL	[Pr](AA)3	OC-6	$D_3$	[1 2 5 4 3 6]	0.3
Nd	TUPYOS	[Nd]a3(AA)3	TCTPR-9	$C_3$	[5 4 3 9 2 6 8 1 7]	6.5
Sm	XAXYAW	[Sm]a(AA)3	COC-7	$C_1$	[1 3 7 2 6 4 5]	0.5
Eu	${\text {MIHNOG}}^*$	[Eu]a2(AA)3	SAPR-8	$C_2$	[1 6 4 2 8 5 3 7]	$-0.6$
Gd	WEWNOB	[Gd](AA)3	OC-6	$D_3$	[1 2 3 4 5 6]	0.0
Tb	BUJCAL	[Tb]a2b2(AA)	OC-6	$C_{2v}$	[1 2 4 6 5 3]	$-0.3$
Dy	ZAXSAS	[Dy](AA)3	OC-6	$D_3$	[1 2 5 4 3 6]	0.0
Ho	PHPRHO10	[Ho]a(AA)3	COC-7	$C_3$	[2 1 3 5 6 7 4]	$-1.1$
Er	DOGKEP	[Er]a2b(AA)3	MFF-9	$C_1$	[2 1 6 4 9 3 5 7 8]	0.4
Tm	MIHPAU	[Tm]a2(AA)3	SAPR-8	$C_2$	[1 4 6 8 2 5 7 3]	$-0.9$
Yb	RENXIR	[Yb](AA)3	OC-6	$D_3$	[1 2 5 4 3 6]	0.0
Lu	POGWEN	[Lu]a3b2	SPY-5	$C_{2v}$	[1 2 4 3 5]	$-1.2$

The last column contains $\Delta \Delta _f H$, the difference in enthalpy of formation between the same compound constructed using the Complex Build algorithm and the crystal structure after both have undergone a geometry optimization by the MOPAC 2016^34^ software using the RM1 Hamiltonian^28–32^ . When the value of $\Delta \Delta _f H$ is negative, the Complex Build starting structure led to a more stable RM1 optimized geometry.

*Crystallographic structure presented one imaginary frequency upon optimization with MOPAC 2016.

As can be seen from Table 5, the absolute differences in the enthalpies of formation for the complexes optimized from the crystallographic structure and the ones optimized from the structures composed by the Complex Build Algorithm often fall below 1 kcal/mol (nine cases), and even in the instances when it goes beyond this range, it almost never reaches values above 10 kcal/mol. This is valid even for complexes with coordination numbers as high as nine.

**Figure 9:**
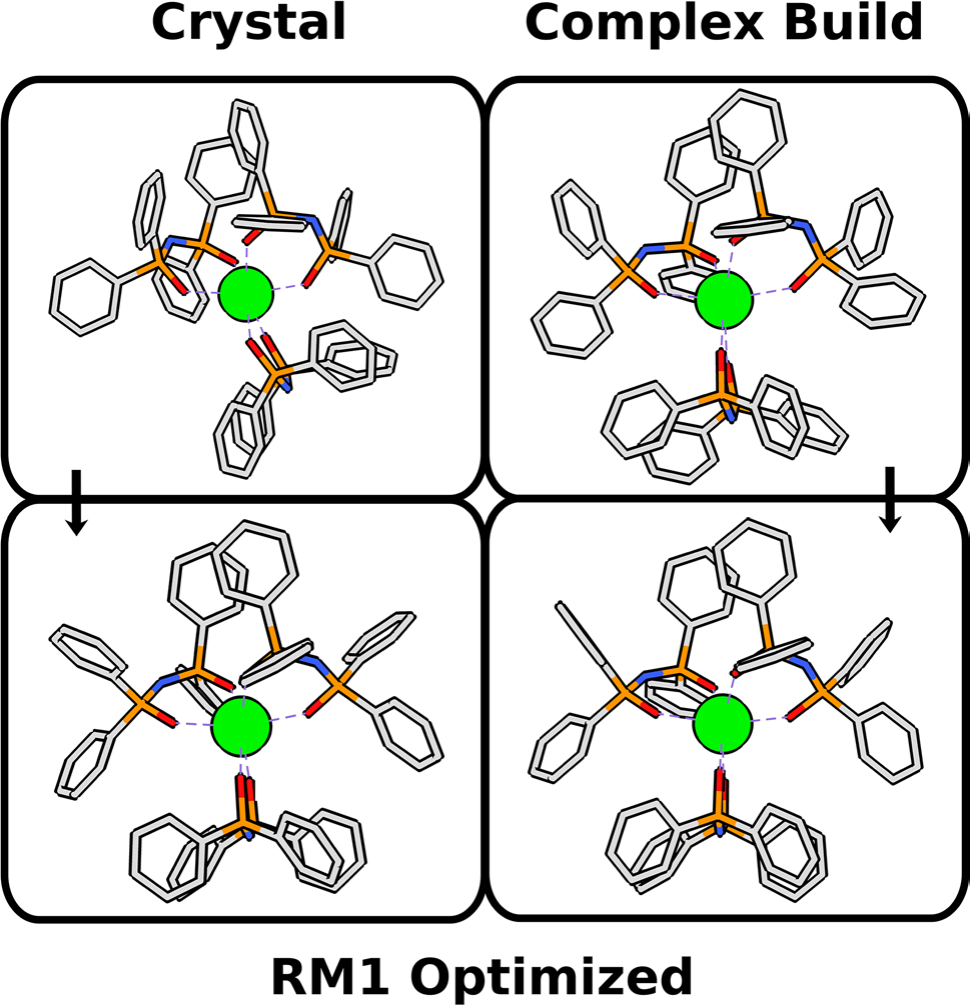
Pr(III) complex tris(tetraphenylimidodiphosphinato)-praseodymium of stereoisomer ID^16^ {[M(AA)3] OC-6 D3 c 6 A [1 2 5 4 3 6]} found in CSD by entry ZAXRUL. On the top left, the crystallographic structure from the Cambridge Crystallographic Data Center, and, on the bottom left, the structure obtained after its RM1 optimization. On the top right, we show the structure composed by the Complex Build algorithm at the same stereoisomer configuration as that of its corresponding crystallographic one; and, on the bottom right, similarly, the structure obtained after its optimization using the RM1 Hamiltonian.

Figure 9 shows a typical case: that of the Pr(III) complex tris(tetraphenylimidodiphosphinato)-praseodymium, of general formula [Pr(AA)3], of coordination number 6 and an octahedron shape of point group $D_3$ and of permutation^16^ [1 2 5 4 3 6] found in CSD by entry ZAXRUL. Each of the three identical symmetric ligands have a charge of $-1$ leading to a neutral complex. Despite the flexibility of the ligands with their large aromatic groups, the structure obtained from Complex Build was very similar to the crystallographic one, both very close to their RM1 optimized geometries. This is an indication that the Complex Build algorithm was capable of successfully handling the several internal ring rotations of the ligands in this compound.

**Figure 10:**
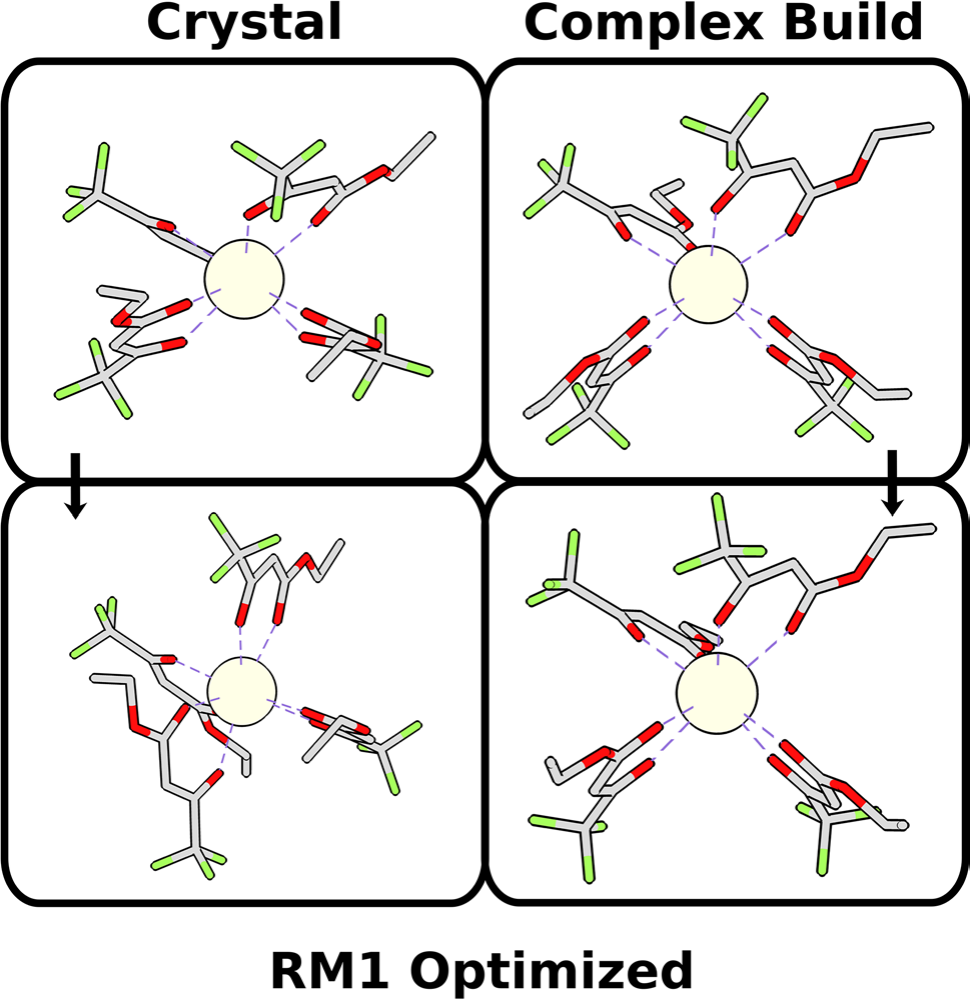
Ce(III) complex tetrakis(1-ethoxy-4,4,4-trifluorobutane-1,3-dionato)-cerium(iii) of stereoisomer ID^16^ {[M(AB)4] SAPR-8 C2 c 2 B [1 4 3 2 8 7 5 6]} found in CSD by entry PUTQAW. On the top left, the crystallographic structure from the Cambridge Crystallographic Data Center, and, on the bottom left, the structure obtained after its RM1 optimization. On the top right, we show the structure composed by the Complex Build algorithm at the same stereoisomer configuration as that of its corresponding crystallographic one; and, on the bottom right, similarly, the structure obtained after its optimization using the RM1 Hamiltonian.

The one and single exception to the pattern of $\Delta \Delta _f H$ in Table 5 is that of complex ammonium tetrakis(1-ethoxy-4,4,4-trifluorobutane-1,3-dionato)-cerium(iii) of general formula [Ce(AA)3], of coordination number 8 of a square antiprism shape of point group $\text {C}_2$ and of permutation^16^ [1 4 3 2 8 7 5 6] found in CSD by entry PUTQAW. The four identical symmetric ligands, each have a charge of $-1$, with the complex displaying an overall charge of $-1$. While the RM1 optimization of the Complex Build starting structure retained the original stereochemical configuration, upon optimization of the crystallographic structure with the RM1 Hamiltonian, the output revealed a different stereoisomer, that is, a different compound, as can be seen in Fig. 10 below, which explains the high $\Delta \Delta _f H$ value of 19.5 kcal/mol. This rearrangement is something that might be expected to happen in some cases after a full geometry optimization by a higher level model chemistry.

**Figure 11:**
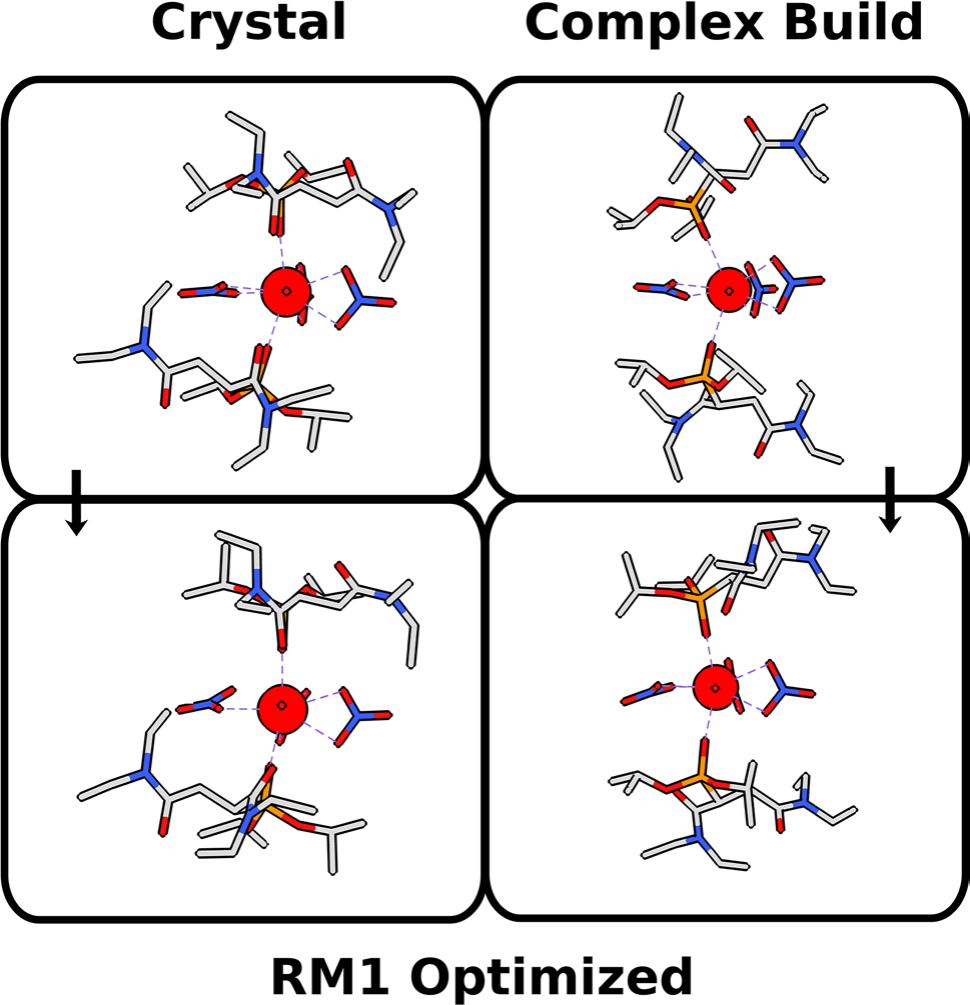
Er(III) complex aqua-bis(di-isopropyl-(1,2-bis(diethylcarbamoyl)-ethyl)-phos phonato)-tris(nitrato-O,O’)-erbium(iii) of stereoisomer ID^16^ {[Ma2b(AA)3] MFF-9 C1 c 1 A [2 1 6 4 9 3 5 7 8]} found in CSD by entry DOGKEP. Ligands depicted above and below the metallic centers in the images are large neutral monodentates, with very long, branched and flexible alkyl moieties. These large groups generate images in which the Erbium ion is depicted slightly off the center, something that makes a comparison among the structures a less straightforward affair. We chose to depict this particular orientation, even though it is not immediately obvious that the larger ligands are monodentates. The little circle in front of the erbium atom is the oxygen atom of a coordinated water. Since the two larger ligands and the water are neutral, and each nitrate has a $-1$ charge, then the complex is neutral.

An example of a complex with very flexible ligands would be that of the erbium complex aqua-bis(di-isopropyl-(1,2-bis(diethylcarbamoyl)-ethyl)-phosphonate)-tris(nitrato-O,O’)-erbium(iii) of general formula Er[a2b(AA)3] of coordination number 9 with a muffin shape, point group ${\text {C}}_1$, and of permutation^16^ [2 1 6 4 9 3 5 7 8] found in CSD by entry DOGKEP. Figure 11 presents the results for this complex. The ligands depicted above and below the metallic centers in the images are the large neutral monodentate di-isopropyl-(1,2-bis(diethylcarbamoyl)-ethyl)-phosphonate ligands, with very long branched and flexible alkyl moieties. These large groups generate images in which the erbium trication is depicted slightly off center, something that makes a comparison among the structures less straightforward. We chose to depict this particular orientation, even though it is not immediately obvious that the larger ligands are monodentates. The little circle in front of the erbium atom is the oxygen atom of a coordinated water. Since the two larger ligands and the water are neutral, and each nitrate has a $-1$ charge, then the complex is overall neutral. These two very flexible ligands present a more difficult case for the Complex Build algorithm because of the likely much more numerous local minima possible. Nevertheless, the final structures obtained from either the crystallographic or Complex Build geometries led to almost the same energy with their values of $\Delta \Delta _f H$ being only 0.4 kcal/mol, indicating a high degree of energetic similarity despite the high flexibility of two of their monodentate ligands.

The “Supplementary Information” contains Figs. S1–S14 with images of the structures, both crystallographic and generated by the Complex Build algorithm, together with their corresponding RM1 fully optimized ones for all 14 complexes of the chosen representative sample.

We point out the fact that none of these 14 structures obtained from the Complex Build algorithm, after RM1^28–32^ quantum chemical full geometry optimizations, displayed normal modes of vibration with negative force constants, indicating that all of them optimized to genuine local minima in the potential energy surface. Of course, it would be unrealistic to expect that every compound constructed by the Complex Build algorithm will necessarily be devoid of imaginary frequencies of normal modes upon full geometry optimization by any higher level model chemistry. However, the Complex Build algorithm was designed for the probability of this occurrence to be as low as possible while still keeping the algorithm executable in short time scales in today’s computers.

## Conclusion

We showed that the Complex Build algorithm, advanced in this article, can reliably produce good quality starting geometries of coordination stereoisomers. This reflects the fact that the ligand-docking algorithm is working as intended and correctly applies the information present in the work by Silva et al.^16^, which contains the permutations of the vertices of the various coordination polyhedra that we use as tools to generate complete sets of coordination isomers. Indeed, the Complex Build geometries for lanthanoid complexes with controlled stereochemistry can now be obtained efficiently and without producing strange outputs once they undergo geometry optimizations by energy-based computational chemistry methodologies.

Central to the Complex Build algorithm is the crowding function: the sum of the steric congestion (a repulsive potential, function of the inverse of the interatomic distances) and the coordination warp function, defined in two distinct mathematical forms: the first one, used when the coordination polyhedron of the complex has a fixed-shape, and, the second one, when such a shape is free. Truly, minimization of the crowding function positions the ligands in a very unobstructed and unclogged manner, so that their degrees of freedom do not hinder or even choke one another, something that would otherwise tend to lead to negative force constants upon further geometry optimizations by more advanced computational model chemistries.

Complex Build employs the Fibonacci lattice for the initialization of free-shape complexes, as well as the ligand-docking procedure for fixed-shape complexes; a combination that allowed us to also include the hinge and wheel angles, leading to a precise balance of structural degrees of freedom for the ligands that is both flexible enough to find a minimum structure but that, at the same time, does not lead to unreasonable, spurious, and obviously wrong geometries.

Moreover, Complex Build also includes coordination chirality recognition. The stochastic pairing between the coordination polyhedron and its mirror image described by Marques et al^25^ as well as the Kearsley superposition algorithm^23^ to test for superimposable pairings (which had been previously advanced for carbon-based stereochemical studies^25^) has been generalized and incorporated to reveal the much richer chirality status of any metal complex coordination polyhedron.

We therefore conclude that implementations of the Complex Build algorithm advanced in this article, as the one presently being developed in our laboratories, will lead to practical high-quality and stereochemically-controlled geometries of metallic complexes for use as starting structures for subsequent molecular modeling protocols.

## Supplementary Information

Supplementary Information.

## Data Availability

The supplementary information file contains Complex Build results for 14 different coordination compounds, each for a different lanthanoid, from La to Lu (except Pr). More information on the Complex Build algorithm, including software download and video tutorials, can be found in https://complexbuild.sparkle.pro.br/.
