# Supplementary material for: The complex build algorithm to set up starting structures of lanthanoid complexes with stereochemical control for molecular modeling
Source: Sci Rep. 2021 Nov 2;11:21493. doi: 10.1038/s41598-021-99525-0 (PMC8564551; doi:10.1038/s41598-021-99525-0)
Supplement: Supplementary file 1 — Supplementary Information. [file 41598_2021_99525_MOESM1_ESM.pdf]

Supplementary Information

The Complex Build Algorithm to Set up Starting Structures of Lanthanoid Complexes with Stereochemical Control for Molecular Modeling

*Gabriel H. L. Munguba, Gabriel A. Urquiza-Carvalho, Frederico T. Silva, Alfredo M. Simas**

Departamento de Química Fundamental, CCEN, Universidade Federal de Pernambuco

50670-901 Recife, Pernambuco, Brazil.

*simas@ufpe.br

Introduction

The figures in this Supplementary Information were made for 14 different coordination compounds, each one for a different lanthanoid, from lanthanum to lutetium, with the sole exception of the exceedingly rare and radioactive element promethium. The compounds were also chosen with the intention of spanning a variety of chemically different ligands, ranging from rigid to very flexible ones - types that constitute a true challenge to the Complex Build algorithm. For this study, we chose to use the RM1 Hamiltonian[1], available in the software MOPAC 2016[2] as a feasible and accurate enough[3] quantum mechanical model to further optimize both the crystallographic and Complex Build structures, used as starting geometries.

For each of the compounds, we show, on the top left, the crystallographic structure from the Cambridge Crystallographic Data Center, and, on the bottom left, the structure obtained after its RM1 optimization.

On the top right, we show the structure of the same compound, using the Complex Build algorithm at the same stereoisomer configuration as that of its corresponding crystallographic one; and, on the bottom right, similarly, the structure obtained after its optimization using the RM1 Hamiltonian.

Each of the figures also displays its corresponding coordination polyhedron shape and point group symbols.

The figure captions also contain further comments on the specifics of each case, highlighting a comparison between the results after using, either the crystallographic, or the Complex Build assembled structures, as starting geometries for further model chemistries calculations.

More information on the Complex Build algorithm, including software download and video tutorials, can be found in https://complexbuild.sparkle.pro.br/.

List of Figures

Figure **S1**: **La(III)** complex tetrakis(N,N,N',N'-tetramethylsuccinamide-O,O')-lanthanum found in CSD entry QAKWEE……………………………………………………………4

Figure **S2: Ce(III)** complex tetrakis(1-ethoxy-4,4,4-trifluorobutane-1,3-dionato)-cerium(iii) found in CSD entry PUTQAW…………………………………………………………..5

Figure **S3: Pr(III)** complex tris(tetraphenylimidodiphosphinato)-praseodymium found in CSD byentry ZAXRUL…………………….………………………………………………6

Figure **S4: Nd(III)** complex tris(N-butylcaprolactam-O)-tris(nitrato-O,O')-neodymium(iii) found in CSD by entry TUPYOS…………..…………………………………………………7

Figure **S5: Sm(III)** complex tris(2,2,6,6-tetramethylheptane-3,5-dionato-O,O')-(4-(dimethyl-amino)pyridine-N)-samarium(iii) found in CSD by entry XAXYAW… ……………………..8

Figure **S6: Eu(III)** complex tris(perfluoroacetylacetonato)-triphenylphosphoryl-europium found in CSD by the entry MIHNOG……………………………………………………...9

Figure **S7: Gd(III)** complex tris(2-phenylamino-4-phenyliminopent-2-ene-N,N')-gadolinium (III) found in CSD entry WEWNOB...……...………………………………………….....10

Figure **S8: Tb(III)** complex dichloro-(1,3-bis(2,6-di-isopropylphenyl)-3-(imino-N)-2-phenyl prop-1-en-1-aminato-N)-bis(tetrahydrofuran)-terbium found in CSD by entry BUJCAL….11

Figure **S9: Dy(III)** complex tris(tetraphenylimidodiphosphinato)-dysprosium found in CSD by entry ZAXSAS ……………………………………………….………………………12

Figure **S10: Ho(III)** complex tris(diphenylpropanedionato)-aqua-holmium found in CSD by entry PHPRHO10……………………………………………………………………….13

Figure **S11: Er(III)** complex aqua-bis(di-isopropyl-(1,2-bis(diethylcarbamoyl)-ethyl)-phos-phonate)-tris(nitrato-O,O')-erbium(iii) found in CSD by entry DOGKEP ……………...…..14

Figure **S12: Tm(III)** complex tris(perfluoroacetylacetonato)-triphenylphosphoryl-thulium found in CSD by entry MIHPAU…………………………………………………………....15

Figure **S13: Yb(III)** complex tris(bipyridyl)-ytterbium found in CSD by entry RENXIR….16

Figure **S14: Lu(III)** complex bis(tetrahydrofuran)-tris(2,6-di-isopropylphenoxy)-lutetium found in CSD by entry POGWEN……………………………………………………..17

Lanthanum

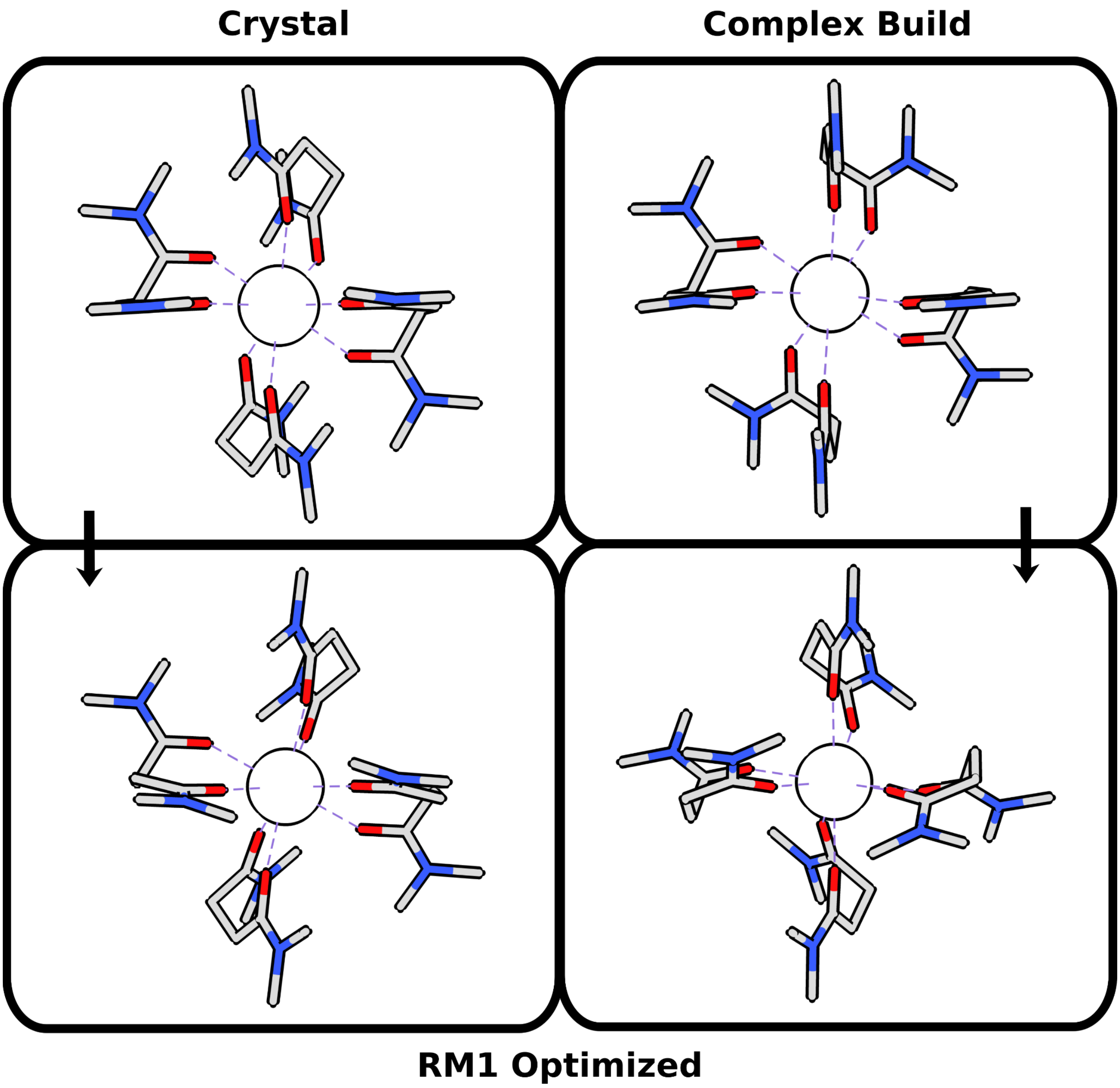

Figure S1: La(III) complex tetrakis(N,N,N',N'-tetramethylsuccinamide-O,O')-lanthanum of stereoisomer ID[4] {[M(AA)4] TDD-8 D2 c 4 B" [1 4 3 2 5 8 7 6]} found in CSD by entry QAKWEE. Please, notice that the four identical bidentate ligands are symmetric, neutral and flexible. The charge of the complex is +3.

Cerium

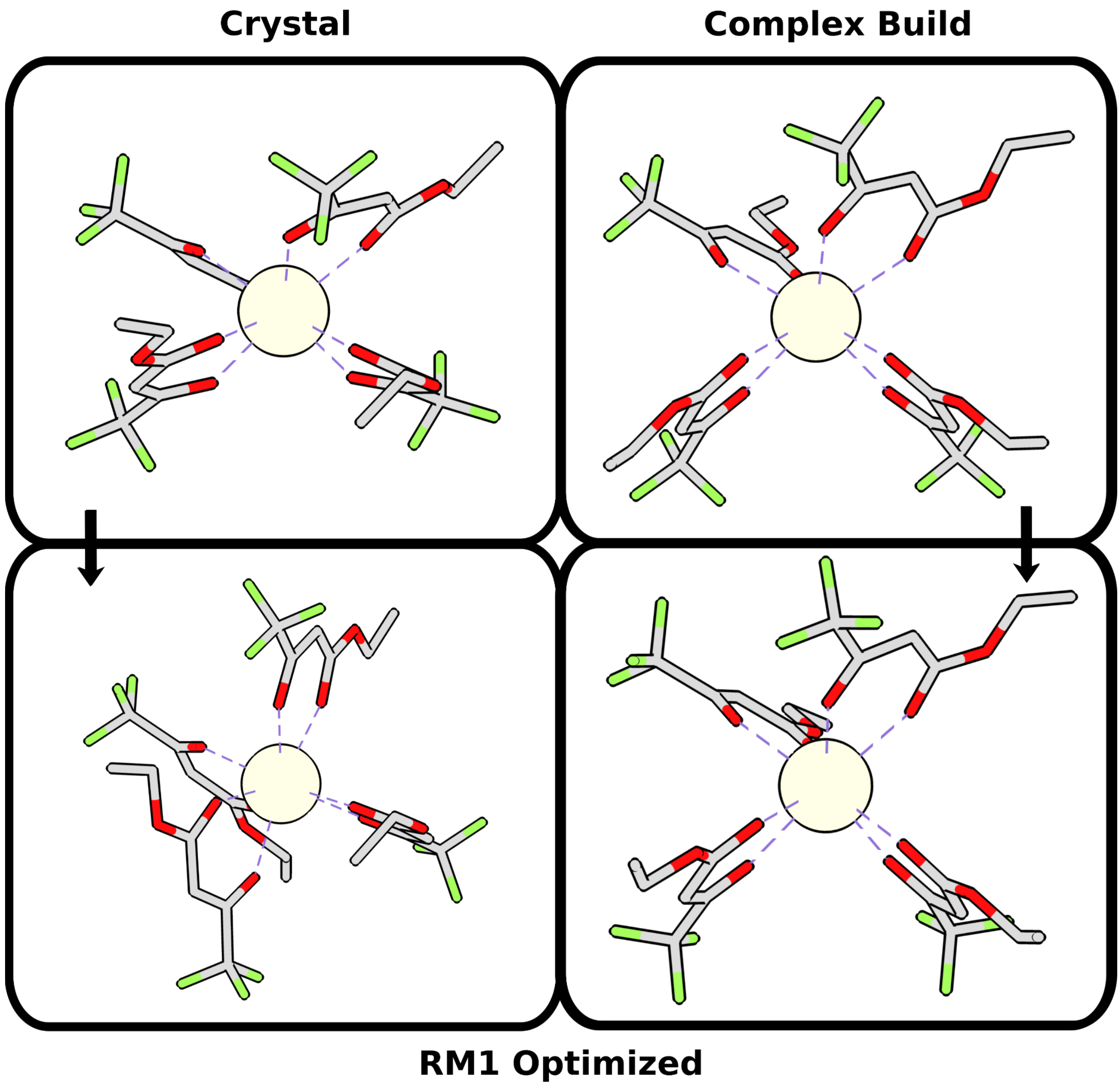

Figure S2: Ce(III) complex tetrakis(1-ethoxy-4,4,4-trifluorobutane-1,3-dionato)-cerium(iii) of stereoisomer ID[4] {[M(AB)4] SAPR-8 C2 c 2 B [1 4 3 2 8 7 5 6]} found in CSD by entry PUTQAW. Upon optimization of the crystallographic structure with the RM1 Hamiltonian, the output revealed a different stereoisomer. On the other hand, RM1 optimization of the Complex Build starting structure retained the original stereochemical configuration. The four identical symmetric ligands, each have a charge of -1, with the complex displaying an overall charge of -1.

Praseodymium

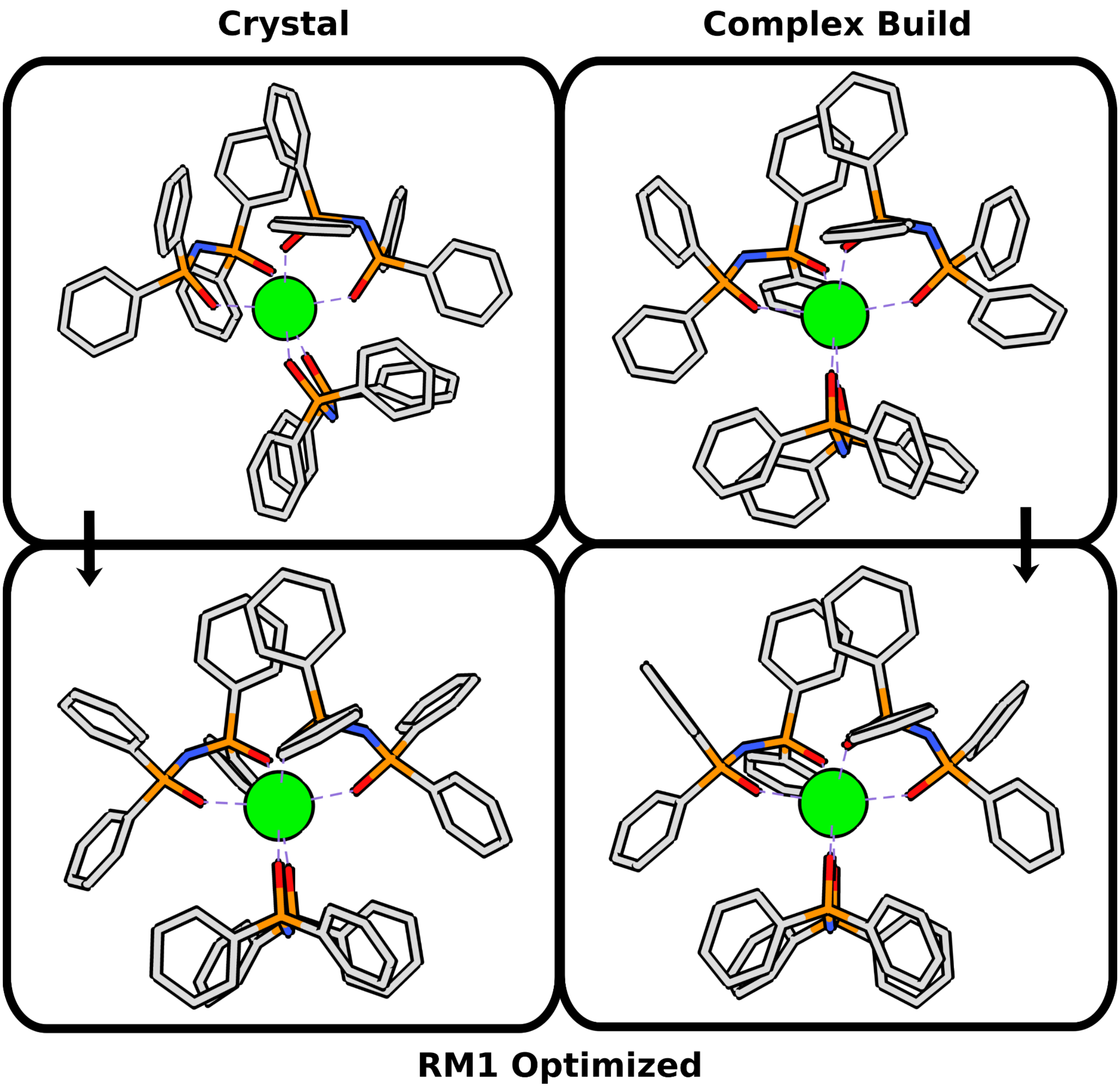

Figure S3: Pr(III) complex tris(tetraphenylimidodiphosphinato)-praseodymium of stereoisomer ID[4] {[M(AA)3] OC-6 D3 c 6 A [1 2 5 4 3 6]} found in CSD by entry ZAXRUL. Despite the flexibility of the ligands with their large aromatic groups, the structure obtained from Complex Build was very similar to the crystallographic one, both very close to their RM1 optimized geometries. Each of the three identical symmetric ligands have a charge of -1 leading to a neutral complex.

Neodymium

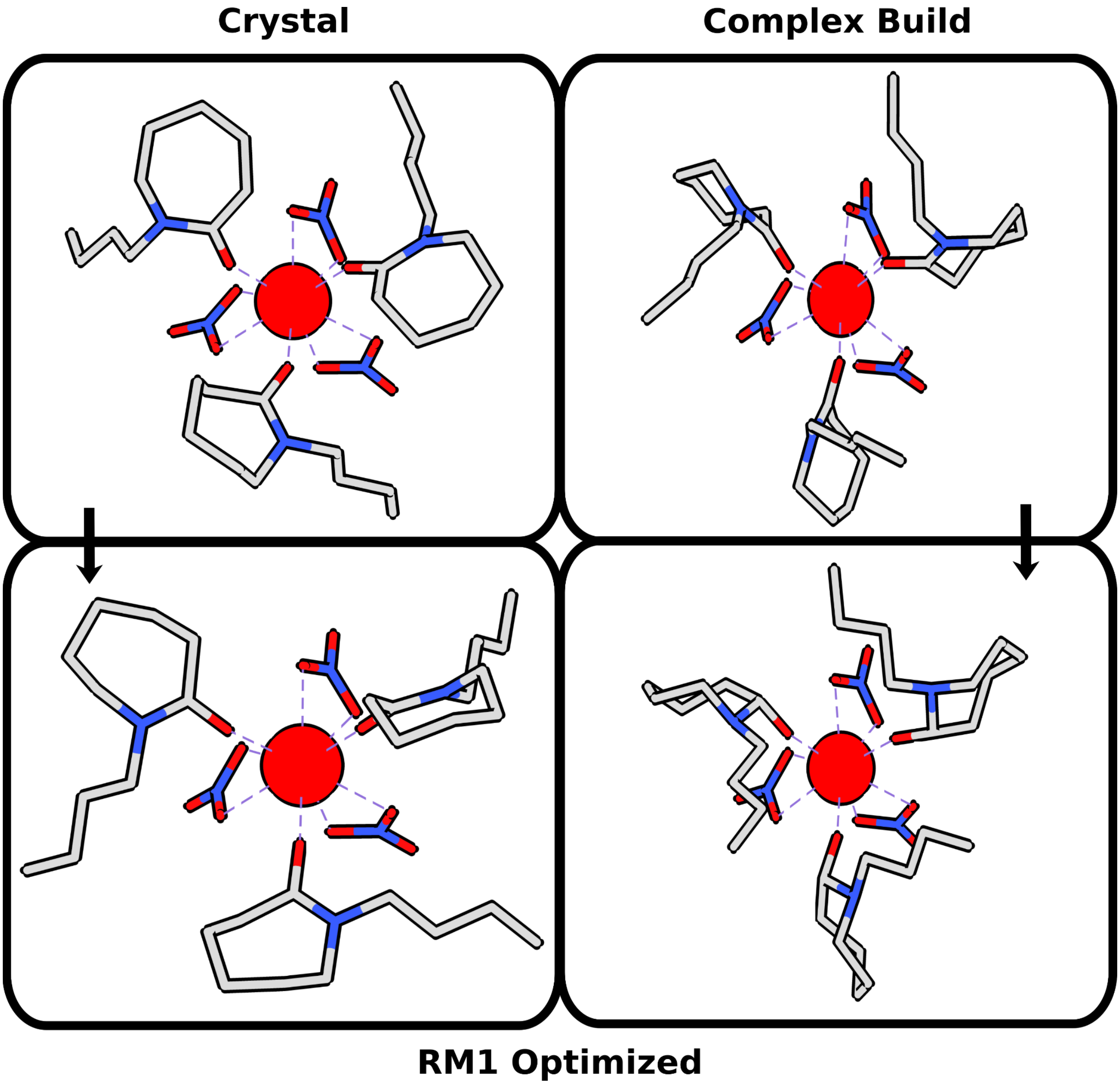

Figure S4: Nd(III) complex tris(N-butylcaprolactam-O)-tris(nitrato-O,O')-neodymium(iii) of stereoisomer ID[4] {[Ma3(AA)3] TCTPR-9 C3 c 3 C [5 4 3 9 2 6 8 1 7]} found in CSD by entry TUPYOS. The three nitrate ions cancel the charge of the trivalent neodymium. The three organic ligands are all identical, neutral, and monodentate. These large ligands with their seven-membered cycloalkyl rings are free to rotate around their respective wheel angles (described in the main article). All this flexibility can lead to structures that may look and are somewhat different from one another, but that nevertheless preserve the coordination stereochemistry in all cases.

Samarium

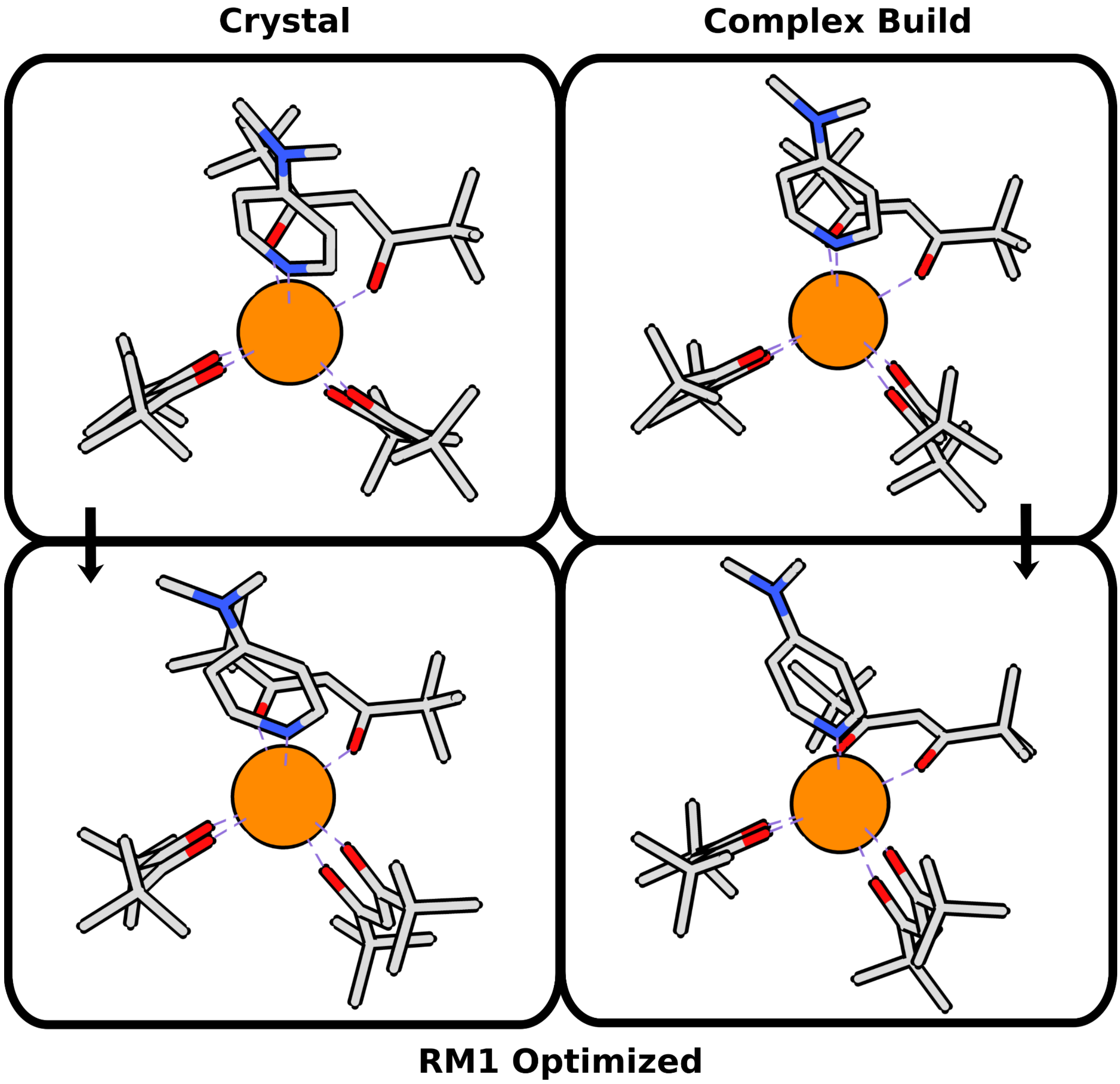

Figure S5: Sm(III) complex tris(2,2,6,6-tetramethylheptane-3,5-dionato-O,O')-(4-(dimethylamino)pyridine-N)-samarium(iii) of stereoisomer ID[4] {[Ma(AA)3] COC-7 C1 c 1 A [1 3 7 2 6 4 5]} found in CSD by entry XAXYAW. The pyridine derivative is neutral and each of the three β-diketonates has a charge of -1, that cancel the +3 charge in the samarium trivalent cation, all leading to a neutral complex. Coordination stereochemistry, and even the overall structural orientation of ligands is evidently preserved in all cases, meaning that the Complex Build algorithm was able to construct a structure very similar to the crystal structure, and that both of these structures were already very close to local minima.

Europium

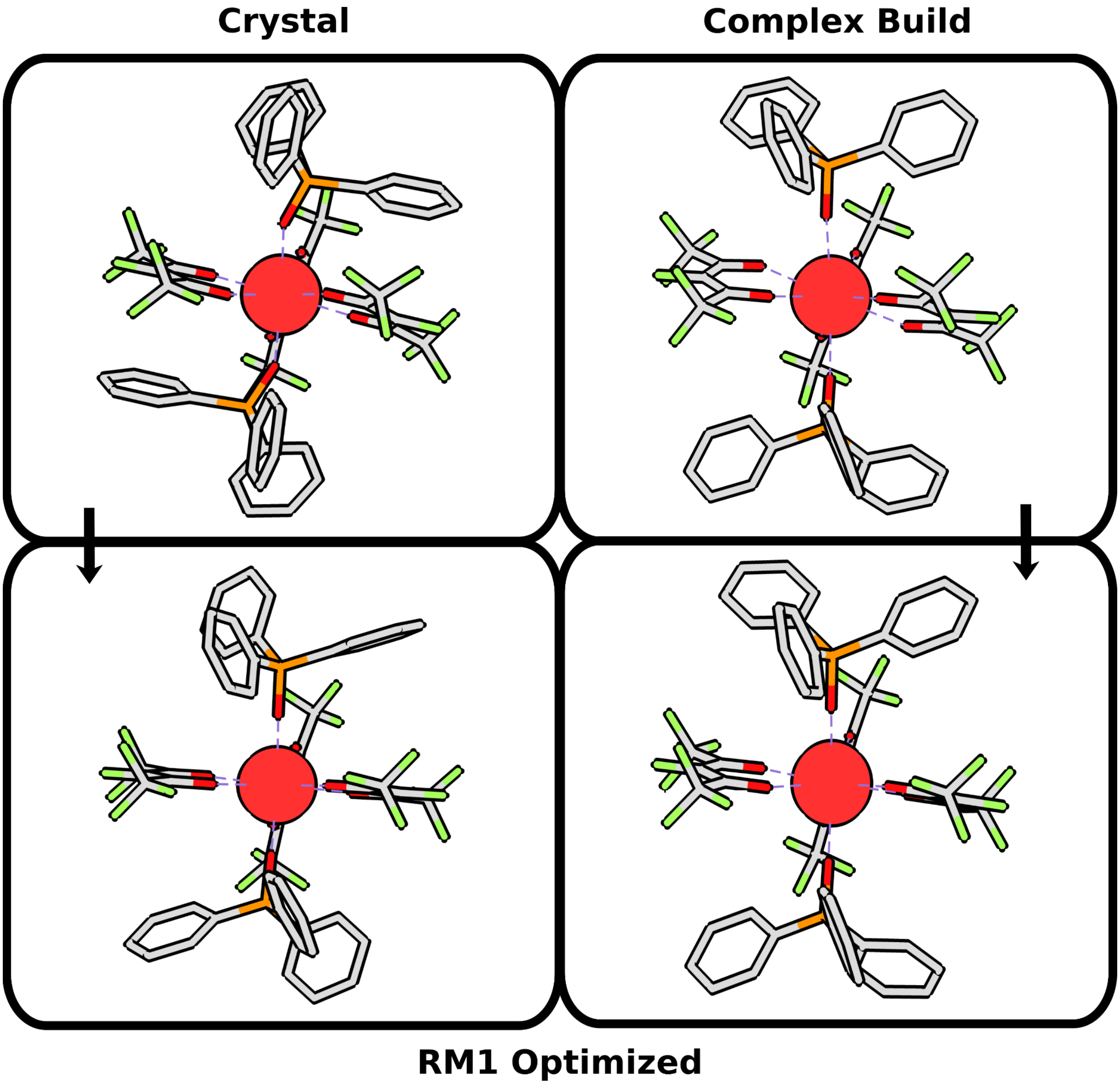

Figure S6: Eu(III) complex tris(perfluoroacetylacetonato)-triphenylphosphoryl-europium of stereoisomer ID[4] {[Ma2(AA)3] SAPR-8 C2 c 2 B [1 6 4 2 8 5 3 7]} found in CSD by the entry MIHNOG. Each of the three fluorinated β-diketonates has a charge of -1 on them, cancelling off the +3 charge in the Europium cation. Since both of the triphenylphosphoryls are neutral, the entire complex has an overall neutral electric charge. Even with many torsionable sigma bonds and ligands with large moieties, the structures are very similar to one another, even the one later optimized with the RM1 method.

Gadolinium

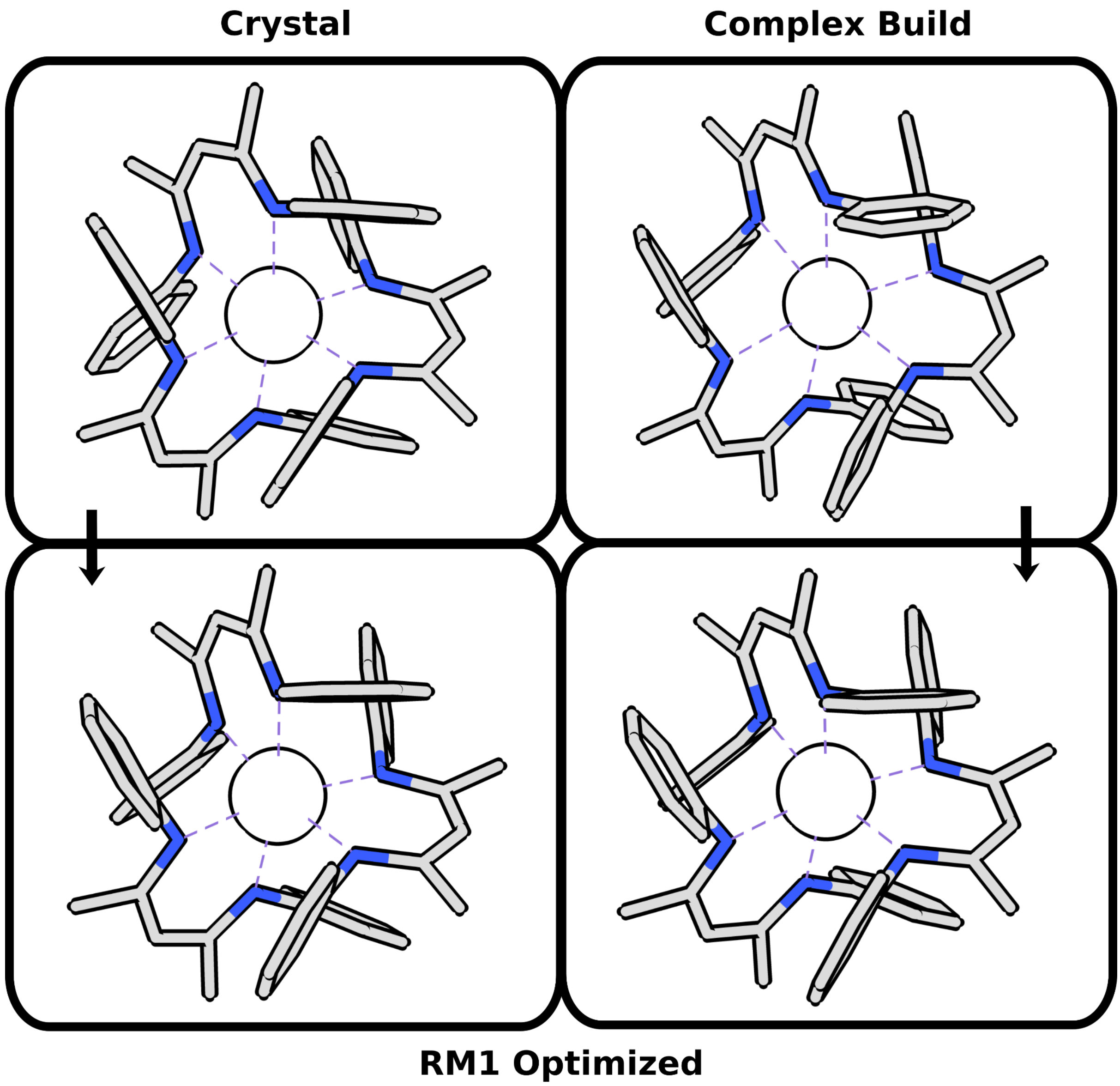

Figure S7: Gd(III) complex tris(2-phenylamino-4-phenyliminopent-2-ene-N,N')-gadolinium(iii) of stereoisomer ID[4] {[M(AA)3] OC-6 D3 c 6 A [1 2 3 4 5 6]} found in CSD entry WEWNOB. Each of the three identical ligands has a charge of -1, leading to a neutral complex. Once again, it is evident that the Complex Build managed to construct the same stereoisomer as the crystal structure and that both of these starting structures led to very similar optimized structures.

Terbium

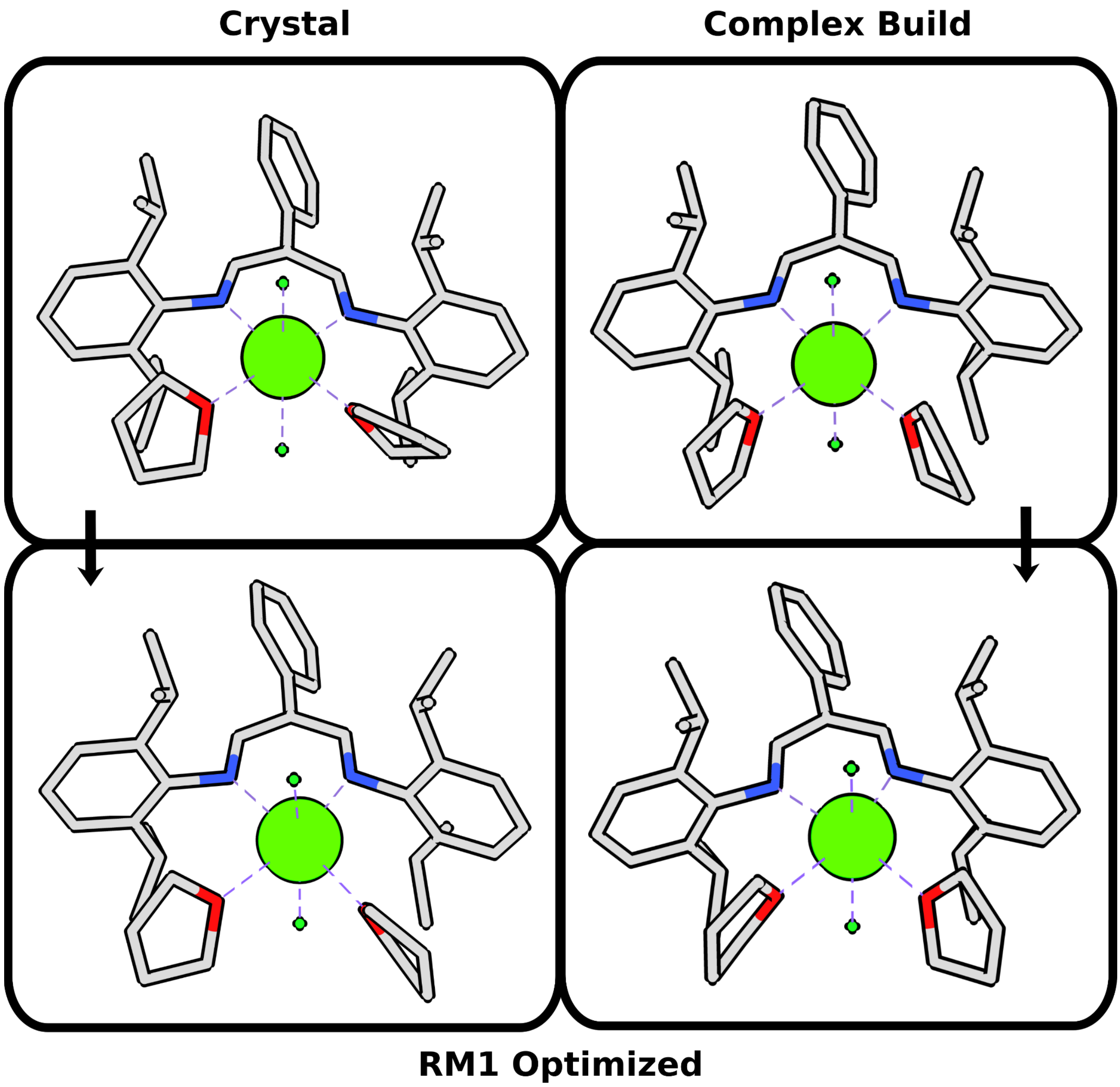

Figure S8: Tb(III) complex dichloro-(1,3-bis(2,6-di-isopropylphenyl)-3-(imino-N)-2-phenyl-prop-1-en-1-aminato-N)-bis(tetrahydrofuran)-terbium(iii) of stereoisomer ID[4] {[Ma2b2(AA)] OC-6 C2v a 2 B [1 2 4 6 5 3]} found in CSD by entry BUJCAL. Each of the two chloride ions, as well as the largest ligand, all have a charge of -1. Since the tetrahydrofurans are neutral, so is the complex. The greatest differences between the crystallographic and the ComplexBuild structures is due to rotations around wheel angles of the monodentate tetrahydrofurans. Notice that even the relative orientations of the isopropyl moieties in the larger ligand are very similar in all four structures.

Dysprosium

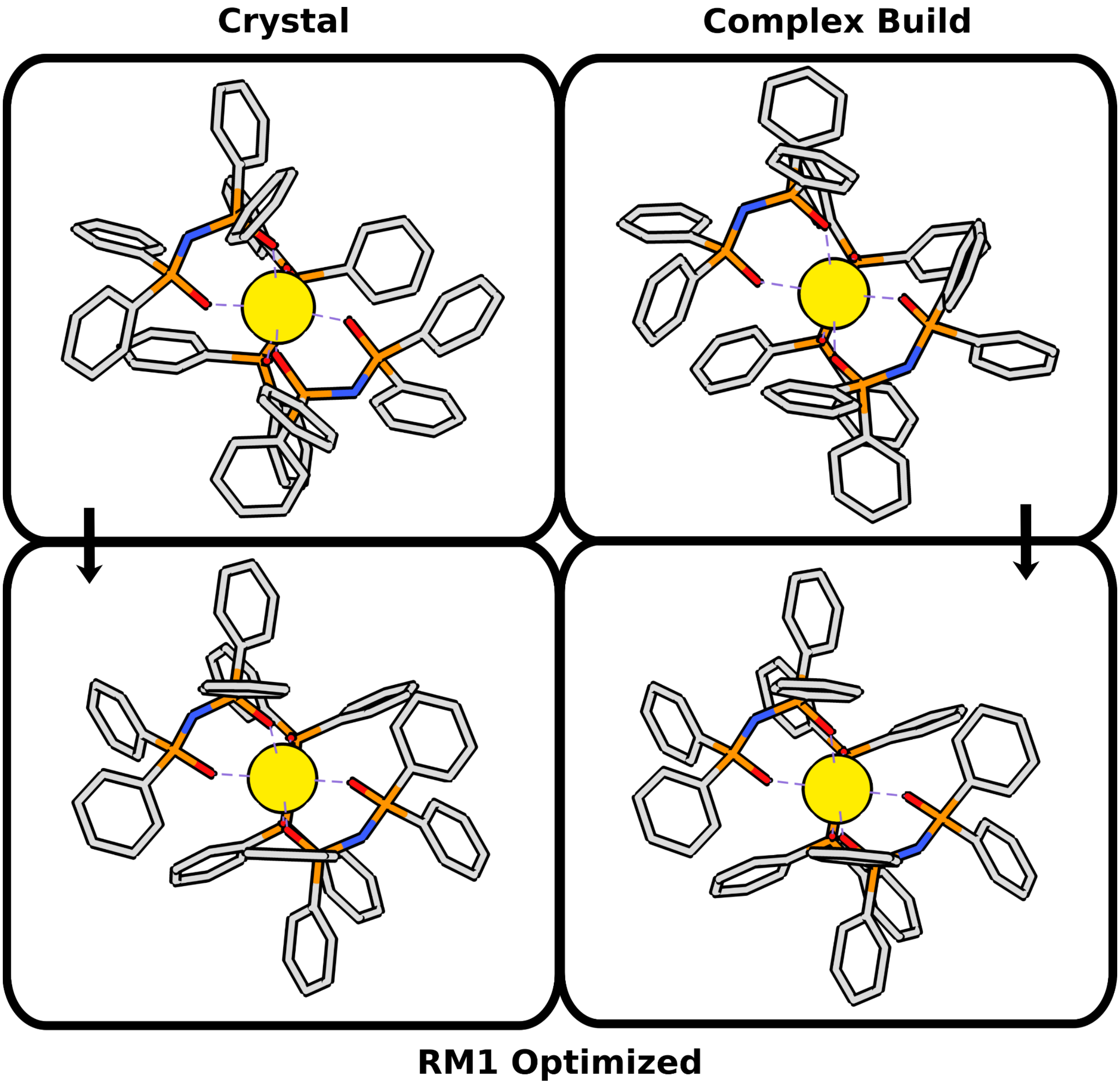

Figure S9: Dy(III) complex tris(tetraphenylimidodiphosphinato)-dysprosium(iii) of stereoisomer ID[4] {[M(AA)3] OC-6 D3 c 6 A [1 2 5 4 3 6]} found in CSD by entry ZAXSAS. Each of the three identical ligands has a charge of -1, all of which cancel the +3 charge of the trivalent dysprosium cation, leading to a neutral complex. Once more, the four structures are evidently quite similar, particularly in the atoms that make up the coordination polyhedron. Both the crystallographic structure and the one obtained from the ComplexBuild optimized to what is essentially the same final geometry.

Holmium

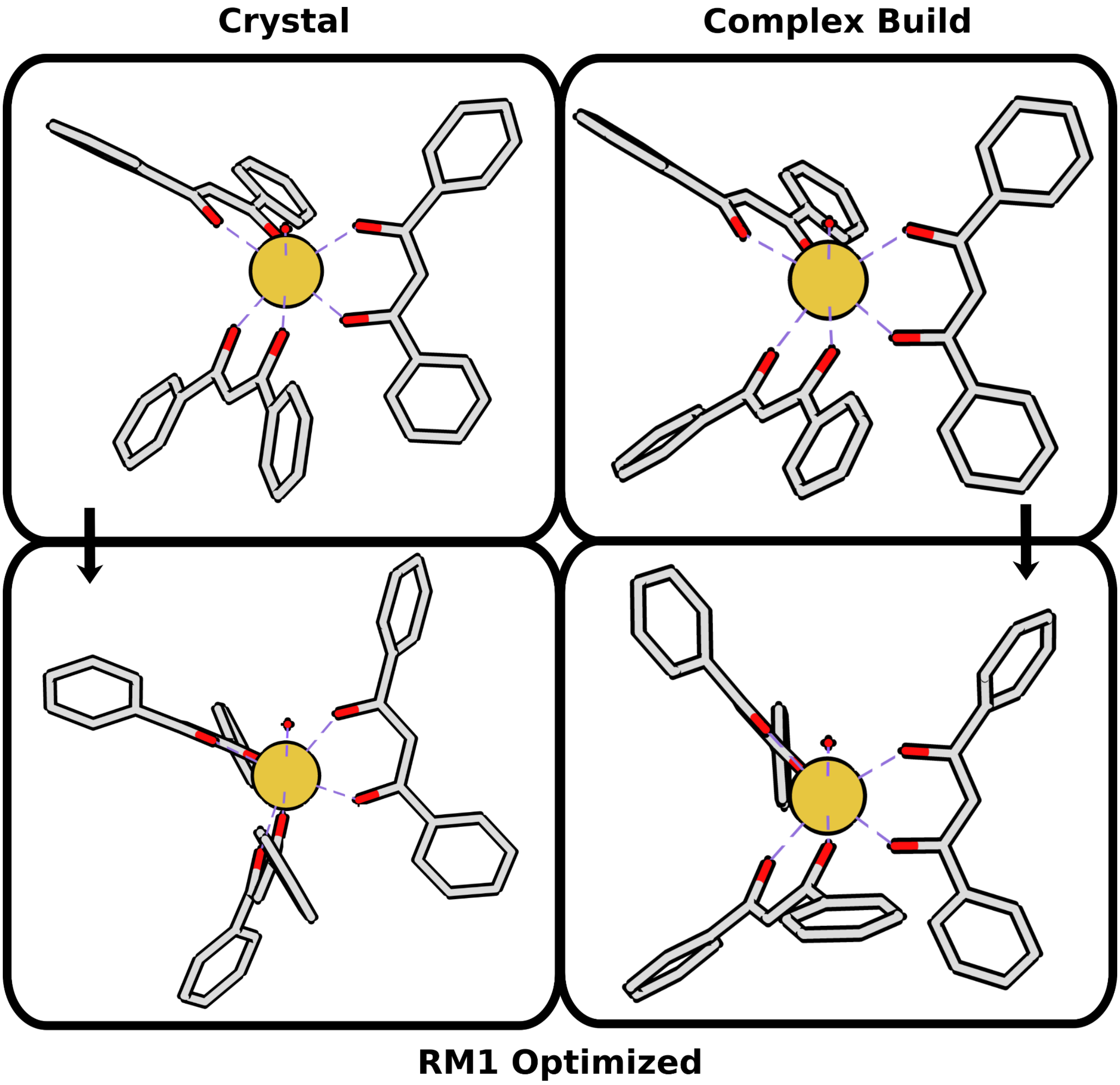

Figure S10: Ho(III) complex tris(diphenylpropanedionato)-aqua-holmium(iii) of stereoisomer ID[4] {[Ma(AA)3] COC-7 C3 c 3 C [2 1 3 5 6 7 4]} found in CSD by entry PHPRHO10. Each of the three β-diketonates has a charge of -1, that cancel the charge of the central holmium trivalent cation. Since the water, the last ligand, is neutral, so is the complex. Whereas most ligands have remained in their original relative positions after the RM1 optimization, the β-diketonate shown in the lower part of the images has changed its coordination orientation in the optimized crystallographic structure. This change led to a different stereoisomer, something that did not happen for the structure obtained from the Complex Build algorithm, used as a starting geometry.

Erbium

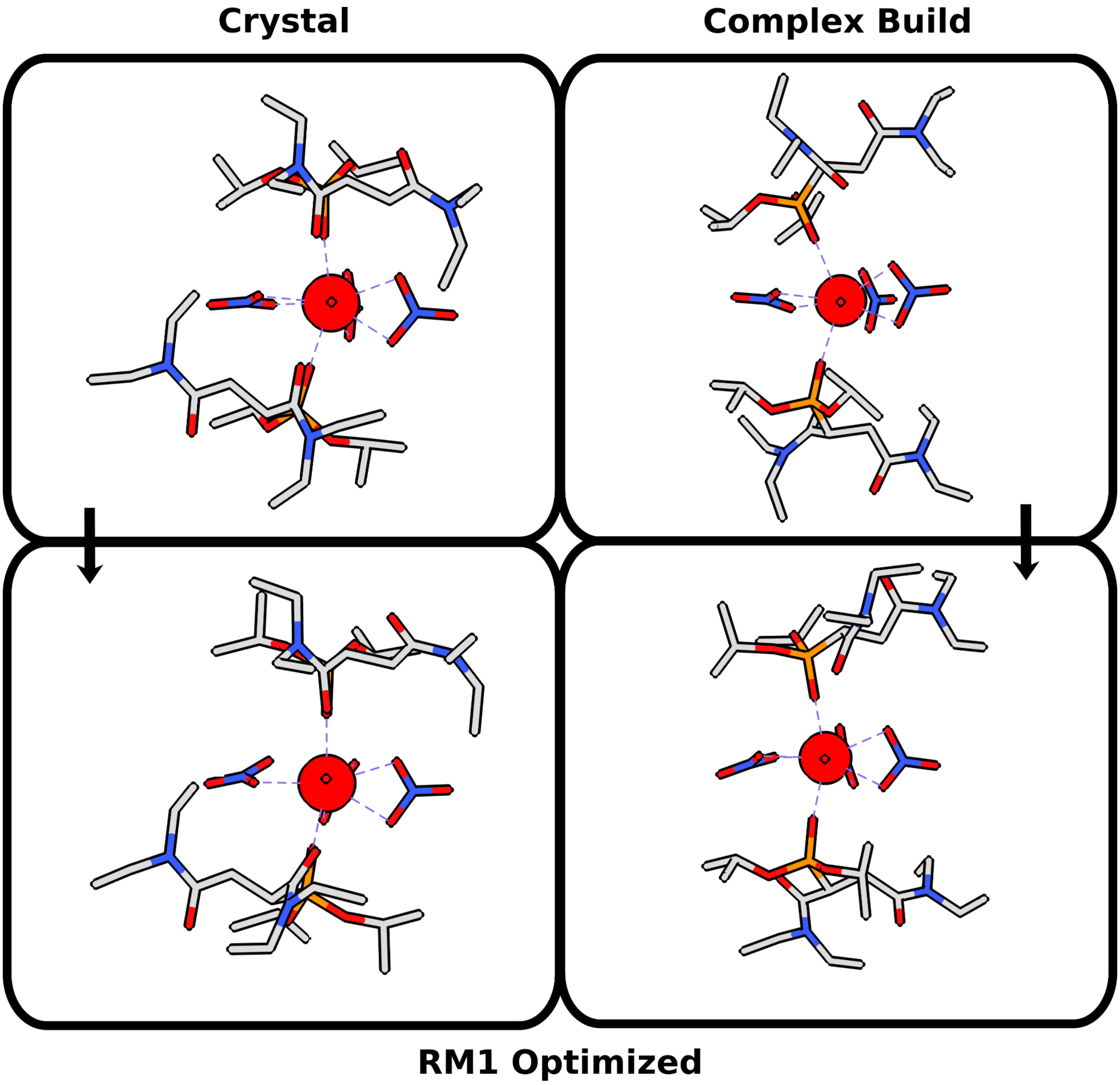

Figure S11: Er(III) complex aqua-bis(di-isopropyl-(1,2-bis(diethylcarbamoyl)-ethyl)-phosphonate)-tris(nitrato-O,O')-erbium(iii) of stereoisomer ID[4] {[Ma2b(AA)3] MFF-9 C1 c 1 A [2 1 6 4 9 3 5 7 8]} found in CSD by entry DOGKEP. Ligands depicted above and below the metallic centers in the images are large neutral monodentates, with very long, branched and flexible alkyl moieties. These large groups generate images in which the Erbium ion is depicted slightly off the center, something that makes a comparison among the structures a less straightforward affair. We chose to depict this particular orientation, even though it is not immediately obvious that the larger ligands are monodentates. The little circle in front of the erbium atom is the oxygen atom of a coordinated water. Since the two larger ligands and the water are neutral, and each nitrate has a -1 charge, then the complex is neutral.

Thulium

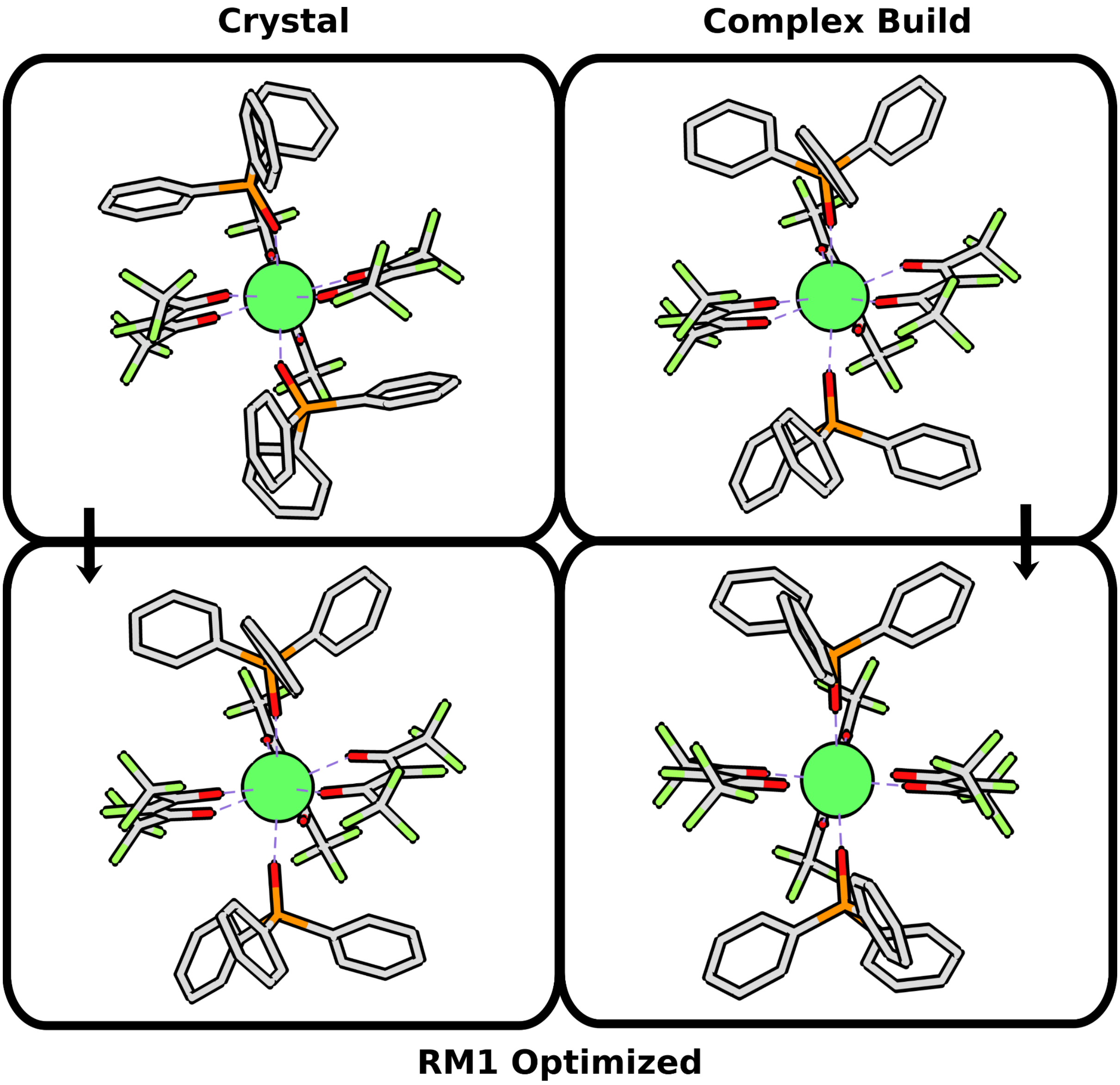

Figure S12: Tm(III) complex tris(perfluoroacetylacetonato)-triphenylphosphoryl-thulium of stereoisomer ID[4] {[Ma2(AA)3] SAPR-8 C2 c 2 B [1 4 6 8 2 5 7 3]} found in CSD by entry MIHPAU. Each of the three β-diketonates has a charge of -1, cancelling the +3 charge in the Europium cation. The remaining ligands are neutral, leading to a neutral complex. It is interesting to note the left-right inversion of the tilt in the bidentate ligand in the back after the RM1 optimization in the ComplexBuild structure. This inversion changes the stereoisomer of the optimized ComplexBuild structure from the original starting structure to its enantiomeric pair, given by the permutation [1 6 4 2 8 5 3 7]. Indeed, in some cases such as this one, minute variations in the starting geometry are enough to veer the optimization path towards either one.

Ytterbium

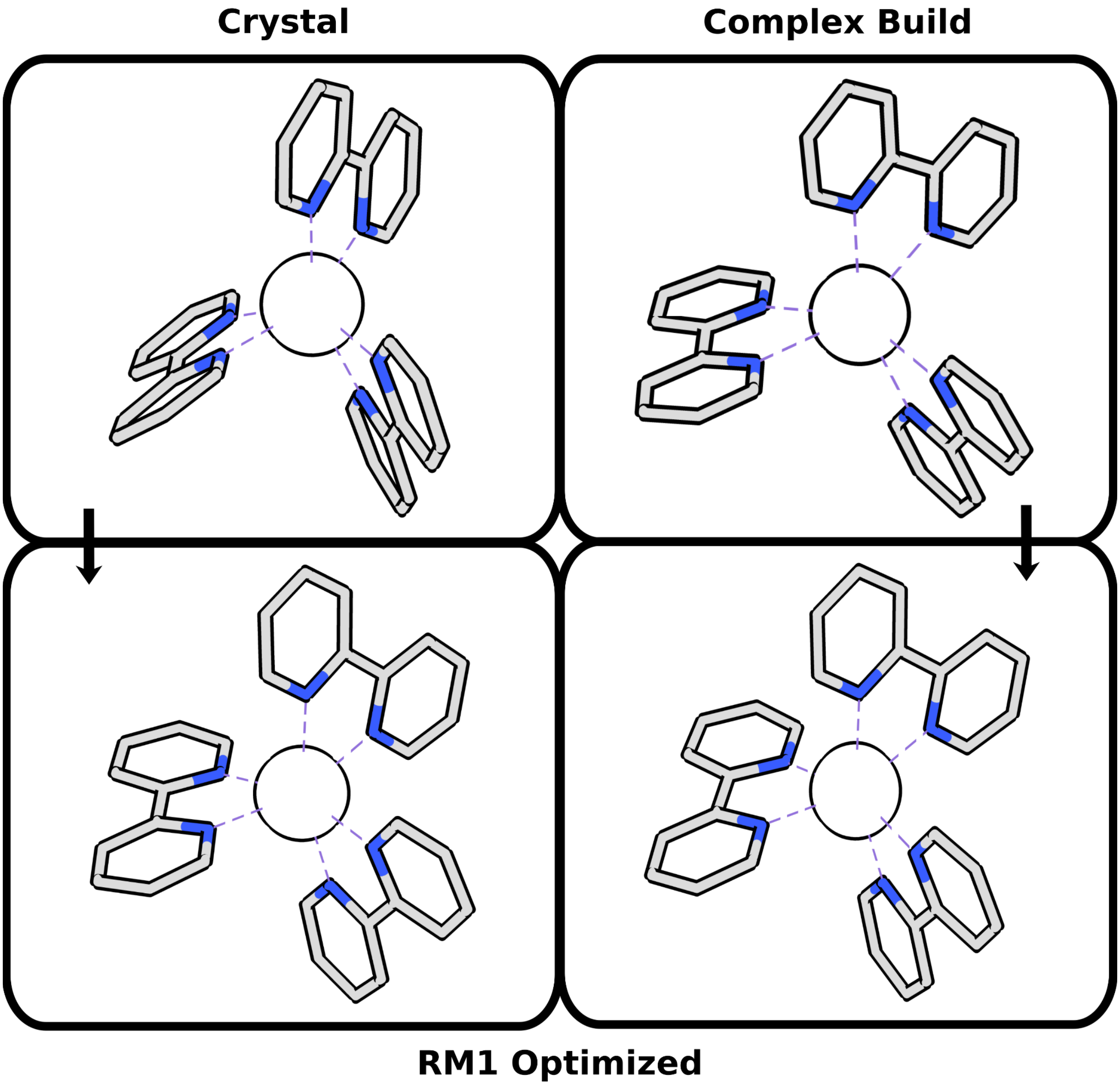

Figure S13: tris(bipyridyl)-ytterbium of stereoisomer ID[4] {[M(AA)3] OC-6 D3 c 6 A [1 2 5 4 3 6]} found in CSD by the entry RENXIR. Since each 2,2-bypiridine is neutral, the complex has a +3 charge. Despite the slight differences in the starting structures, they both converged to the exact same final optimized structure, which was closer to the starting geometry obtained from the Complex Build algorithm.

Lutetium

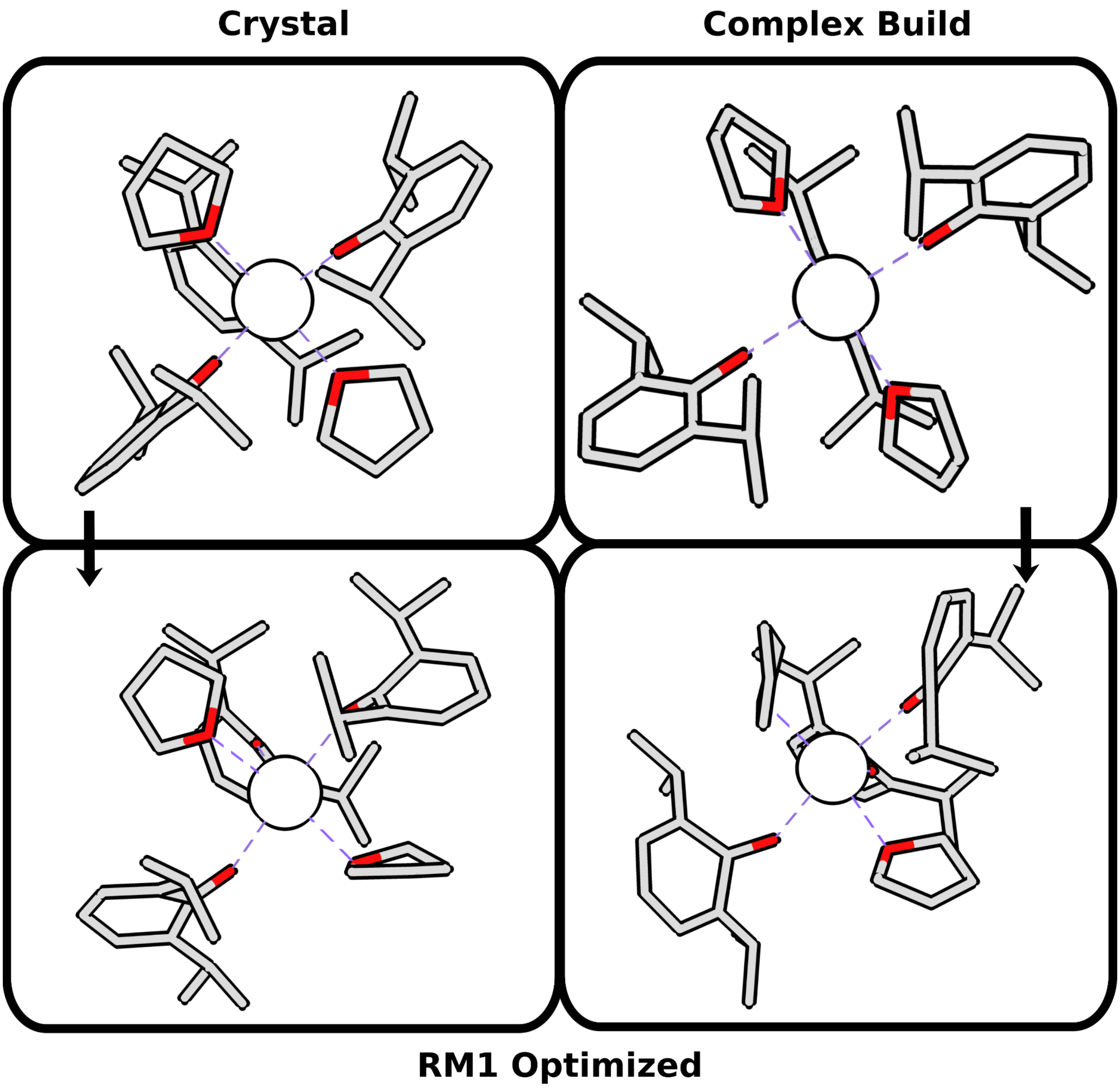

Figure S14: Lu(III) complex bis(tetrahydrofuran)-tris(2,6-di-isopropylphenoxy)-lutetium of stereoisomer ID[4] {[Ma3b2] SPY-5 C2v a 2 B [1 2 4 3 5]} found in CSD by entry POGWEN. Every ligand is neutral, leading to a complex with a net +3 electric charge. Once more, the greatest source of difference between the four structures are the rotations around wheel angles since these are all large monodentate ligands. Nevertheless, both final optimized structures preserved the coordination stereochemistry despite displaying distinct geometric features.

References

1. Filho, M. A. M. *et al.* RM1 Model for the Prediction of Geometries of Complexes of the Trications of Eu, Gd, and Tb. *J. Chem. Theory Comput.* **10**, 3031–3037 (2014).

2. Maia, J. D. C., Dos Anjos Formiga Cabral, L. & Rocha, G. B. GPU algorithms for density matrix methods on MOPAC: linear scaling electronic structure calculations for large molecular systems. *J. Mol. Model.* **26**, 313 (2020).

3. Lima, N., Rocha, G., Freire, R. & Simas, A. RM1 Semiempirical Model: Chemistry, Pharmaceutical Research, Molecular Biology and Materials Science. *Journal of the Brazilian Chemical Society* (2018) doi:10.21577/0103-5053.20180239.

4. Silva, F. T., Lins, S. L. S. & Simas, A. M. Stereoisomerism in Lanthanide Complexes: Enumeration, Chirality, Identification, Random Coordination Ratios. *Inorg. Chem.* **57**, 10557–10567 (2018).
